# Analysis of Cannabinoids in Biological Specimens: An Update

**DOI:** 10.3390/ijerph20032312

**Published:** 2023-01-28

**Authors:** Mónica Antunes, Mário Barroso, Eugenia Gallardo

**Affiliations:** 1 CICS-UBI—Health Sciences Research Centre, University of Beira Interior, Avenida Infante D. Henrique, 6201-506 Covilha, Portugal; 2 Serviço de Química e Toxicologia Forenses, Instituto Nacional de Medicina Legal e Ciências Forenses, Delegação do Sul, Rua Manuel Bento de Sousa 3, 1169-201 Lisboa, Portugal; 3 Laboratório de Fármaco-Toxicologia, UBIMedical, Universidade da Beira Interior, EM506, 6200-284 Covilha, Portugal

**Keywords:** cannabinoids, biological specimens, analysis, toxicology

## Abstract

Cannabinoids are still the most consumed drugs of abuse worldwide. Despite being considered less harmful to human health, particularly if compared with opiates or cocaine, cannabis consumption has important medico-legal and public health consequences. For this reason, the development and optimization of sensitive analytical methods that allow the determination of these compounds in different biological specimens is important, involving relevant efforts from laboratories. This paper will discuss cannabis consumption; toxicokinetics, the most detected compounds in biological samples; and characteristics of the latter. In addition, a comprehensive review of extraction methods and analytical tools available for cannabinoid detection in selected biological specimens will be reviewed. Important issues such as pitfalls and cut-off values will be considered.

## 1. Introduction

*Cannabis*, widely known as marijuana or hemp, is a genus in the family *Cannabinaceae* that grows in temperate and tropical areas such as Eastern and Central Asia. *Cannabis sativa* L., one of the subspecies of *Cannabis*, is the most controversial plant in the world. According to the United Nations Office on Drugs and Crime (UNODC), cannabis is the most popular illicit drug of the century, being consumed in similar quantities as legal drugs such as tobacco, alcohol, and caffeine [1]. It is the most widespread illicit drug in the world [2].

The medicinal properties of this plant have been known for centuries [2], but recently there has been an increased interest in the therapeutic properties of its main active secondary metabolites [1]. Medicinal cannabis has been used in the treatment of chronic pain, cancer pain, depression, sleep disturbances and anxiety, and neurological disorders. It has been widely used in cases of neurodegenerative conditions such as Parkinson’s disease, Alzheimer’s disease, and multiple sclerosis, as well as in cases of post-traumatic stress disorder, Tourette’s syndrome, epilepsy, arthritis, and nausea and vomiting due to chemotherapy, and as an appetite stimulator in HIV/AIDS patients. Cannabinoids have also gained interest in the dermatologic field [1,2]. This recent interest, however, has affected aspects such as public health and production, use, and sale of cannabis plants, which has led to legislation changes in several countries. The legalization and ethical implications of this plant are polarizing topics, so the use of this plant for medicinal purposes is a complex matter, particularly considering the psychotropic effects it also comprises [1].

**Figure 1:**
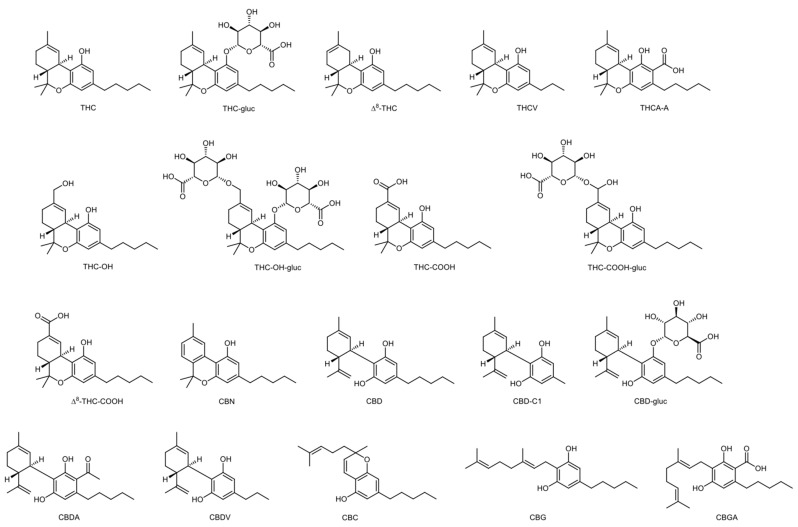
Naturally occurring cannabinoids and metabolites.

There are more than five hundred forty-five different compounds in a *Cannabis* plant. Considering cannabinoids, over one hundred different compounds have been identified so far, belonging to different families [1]. Δ^9^-tetrahydrocannabinol (THC) is the primary psychoactive analyte of cannabis [3]. There are four different stereoisomers, but only the (-)-trans form exists naturally [1]. Its metabolism takes place mostly in the liver [3] but also in other tissues such as the brain, small intestine, heart, and lungs [1,4,5]. The main pathway involves hydroxylation of THC into the phase I active metabolite 1-hydroxy-Δ^9^-tetrahydrocannabinol (THC-OH), which is then oxidized into the phase I inactive metabolite 11-nor-9-carboxy-Δ^9^-tetrahydrocannabinol (THC-COOH) [3,6,7]. Then, THC and metabolites undergo phase II biotransformation to glucuronide conjugates [7]. In the plant, THC is formed by the decarboxylation of Δ^9^-tetrahydrocannabinolic acid A (THCA-A) [3], and, when cannabis is smoked, combustion converts THCA to THC. Cannabinol (CBN) and cannabidiol (CBD) are also important compounds that can be found in cannabis and have no psychotropic properties, showcasing different pharmacological effects than THC [1]. Δ^8^-tetrahydrocannabinol (Δ^8^-THC) is usually present in the plant in low concentrations, but fresh marijuana’s THC content can have up to 10% of this cannabinoid [8]. Figure 1 shows the chemical structures of the main cannabinoids and cannabinoid glucuronides.

Cannabis is widely known for the feeling of relaxation it provides to users [1]. The most common effects of cannabis are euphoria and physical inertia—characterized by signs of ataxia, dysarthria, and incoordination. This drug also affects memory, cognition, motor function, and psychomotor performance, and it can affect the speed of thought and reaction time. Additionally, after cannabis intake, users experience an increase in blood pressure. The symptoms appear after a few minutes and can last for several hours [9,10].

Considering these effects, driving under the influence of cannabis is a major concern. Other than alcohol, cannabis is the most common drug to be detected in driving under the influence of drugs (DUID) cases [11], and its use poses an even greater risk when used concomitantly with other drugs, which is often the case [12]. The risk is higher when cannabis is consumed with alcohol, since their effects on the psychomotor impairment are additive [9,10]. Cannabis is also recurrently associated with cases of occupational accidents, child custody, or drug-facilitated crimes [2].

*Cannabis sativa* can be consumed in several different preparations, namely marijuana, hashish, hash oil, charas, dagga, and bhang [1,6]. There are different chemotypes of this drug that should be considered, that differ according to the amount of THC present. The fiber type (hemp) has less than 0.3% of THC and is cultivated for the textile and food industries, thus being legal in several countries. The intermediate type has 0.3–1.0% of THC. The drug type (marijuana) has 1.0–20% of THC. This amount of the psychotropic compound brands marijuana as an illicit drug in several countries, and, as such, cultivation of the plant is prohibited [13].

Cannabis consumption can be carried out in multiple ways, but most recreational users use it via the airways—by smoking or vaporization [4]. These methods allow a fast and efficient passage from the lungs to the brain [14], and THC and CBD appear in plasma just a few seconds after inhalation [1,5]. Oral administration is also quite common for both therapeutic and recreational purposes since there is no formation of harmful compounds during consumption. The intake can be carried out in the form of capsules, food, or cannabis-infused drinks [14]. Oral mucosal delivery and sublingual, dermal, rectal, and ophthalmic administrations have also been reported, mainly for therapeutic applications [4,5].

Cannabis, cannabis resin and extracts, and tinctures of cannabis were included in the list of drugs in Schedule I of the United Nations Single Convention on Narcotic Drugs in 1961, which classifies cannabis as a drug, and, thus, subjects it to all measures of control applicable to other drugs [15]. This document was the reason why cannabis’s use for medical purposes dropped in the twentieth century [1].

Over 83 million European adults are estimated to have consumed illicit drugs at some point in their lives, and a value of 78.6 million is reported for the use of cannabis alone. These statistical data vary greatly depending on the country, from 4.3% in Malta to 44.8% in France. Cannabis is still the most consumed drug on the continent, since over 22 million European adults reported using in 2021 [12], probably because it is so easily acquired, and there is low prevalence of dependence situations [1].

In the 1990s, the medicinal use of cannabis products was legalized in several states of the United States of America. Canada followed in 1999, and, since then, many other countries have implemented this condition as well [1]. Most EU countries already allow the medicinal use of cannabinoids, even though the products that are permitted and the regulatory frameworks may be different depending on the country [12].

Considering the growing market for cannabis and the several different types of products that are now easily available, analytical methodologies are constantly updated and studied to monitor this growth. A wide number of cannabinoids and their metabolites can be analysed in several different samples to access cannabis consumption and use. Numerous different cannabinoids are now analysed in the routines of toxicology laboratories around the world. These analyses are of extreme importance to differentiate between licit or illicit consumption and to determine the concentrations of active components [1]. Different aspects should be considered when studying cannabinoids’ analysis: samples, clean-up methods, and analytical instrumentation that should be applied.

Cannabinoids can be analysed in both biological and non-biological samples, and it is extremely important to choose the right matrix to perform an analysis, based on its purpose [1]. Different biological matrices provide different information about time and extent of use [2].

Sample preparation is necessary prior to analysis considering the complexity of most matrices. This procedure includes homogenization of the samples, extraction of the analytes, and a clean-up step to remove interferences. Liquid-liquid extraction (LLE) and solid-phase extraction (SPE) are the most widely used extraction methodologies for routine cannabinoid determination, although modern microextraction techniques have been emerging in the last few years since they are cheaper, faster, require fewer amounts of sample and organic solvents, and have good extraction efficiencies [1]. It is relevant to point out that the pH should be adjusted prior to LLE, considering the variation of the chemical properties of various cannabinoids (THC is neutral and THC-COOH is acidic, for example) [6]. The main advantage of SPE is the great cannabinoid recoveries, but these methodologies are also complicated, time consuming, and difficult to automate and require large amounts of sample and organic solvents [16]. In general, polar solvents are best suited to extract cannabinoids, but it is also acceptable to use a mixture of polar and non-polar solvents (such as n-hexane and ethanol). Acetone (a less polar solvent) is also a good option to extract THC, since it extracts fewer sugars and polysaccharides than methanol [17,18,19].

Usually, cannabinoids are first detected through immunoassay testing, with methods such as enzyme multiplied immunoassay technique (EMIT), enzyme-linked immunosorbent assay (ELISA) [20], fluorescence polarization, and radioimmunoassay. After these preliminary methods have been employed, confirmatory analysis should be carried out, since immunoassays have been associated with false negative and false positive results that occur due to structurally similar compounds that can be recognised by the antibodies, adulterants that affect the pH, detergents, or other surfactants [21].

Another technique used for cannabinoids’ screening is thin-layer chromatography (TLC). This technique can detect both the neutral and the acidic forms of several different cannabinoids in one assay, and several samples can be analysed simultaneously. Thus, it is an extremely cheap and high-throughput method for the screening of these compounds in crude specimens [22,23,24], but not in human biological matrices, however.

Most toxicology laboratories use gas chromatography coupled to mass spectrometry (GC-MS) methodologies for confirmatory analysis of cannabinoids in matrices such as hair and oral fluid (OF). However, GC methodologies are associated with difficulties regarding identification and quantification of acidic cannabinoids such as THC-COOH, THCA, cannabigerolic acid (CBGA), and cannabidiolic acid (CBDA), since they are decarboxylated into their neutral forms during analysis [25,26]. To surpass this problem, derivatization techniques can be employed [27]. Lately, there has been a rising interest in the use of liquid chromatography coupled to mass spectrometry (LC-MS) for confirmatory analysis, considering its high sensitivity, selectivity, and wider applicability. Unlike what happens with GC-MS methodologies, LC-MS does not require time-consuming and expensive derivatization steps to reach similar sensitivity [3,28]. LC methodologies are appropriate to analyze the native composition of the cannabis plant [25], especially considering the low concentrations in which some cannabinoids are present in the samples. Chromatographic techniques with tandem mass spectrometry (MS/MS) and time-of-flight (TOF) capabilities can simultaneously identify a wide range of analytes in a single analysis, which is a significant advantage when dealing with small sample volumes [29].

This review gathers most of the analytical methodologies developed in the last years to identify and quantify different cannabinoids in both conventional and non-conventional biological matrices. Considering that each biological sample has a plethora of different physiological and chemical properties that can modify xenobiotics’ disposition [11], drug test results may differ when different matrices are analysed. Thus, it is extremely important to know the different behaviours certain drugs may present in different matrices. Several review papers have been published concerning this matter, but they tend to focus either on the study of a specific biological sample in analyzing different xenobiotics [11,30,31,32], or in a specific analytical technique [2,33,34]. This review fills this gap by showcasing a comprehensive comparison between the most-used techniques for cannabinoid analysis in different samples. Karschner et al. [35] published a similar work in 2020, so our review serves as an updated version on this matter, since analytical toxicology is constantly evolving.

## 2. Materials and Methods

In this work, three different search engines were used for systematic research: Medline, ISI Web of Knowledge and Google Scholar. Keywords for search were “cannabis”, “cannabinoids”, “analytical”, “blood”, “plasma”, “serum”, “urine”, “oral fluid”, “hair”, “sweat”, “breath”, “nails”, “cerumen”, “meconium”, “amniotic fluid”, “placenta”, “breast milk”, “bile”, “vitreous humor”, and “pericardial fluid”. Due to the high number of manuscripts concerning the determination of cannabinoids in whole blood, plasma, serum, urine, OF, and hair, only the last three years were included (2020–2022). However, concerning other specimens, the last five years were included (2018–2022) because of the low number of papers available.

Regarding inclusion criteria, only studies on biological matrices concerning natural cannabinoids and written in English were considered. Furthermore, only analytical methods that were developed in the studied time frame were selected. Studies on synthetic cannabinoids, studies on animals, and studies on non-biological materials were excluded. Recent papers that used analytical methods developed in previous years were reviewed and included only if relevant in the context of this review. For hair, urine, and OF, only analytical methodologies capable of detecting the cut-off concentrations or lower were selected.

The articles were independently selected by two of the authors for each class of biological specimens to determine their relevance in the context of the current review. Initially, 1927 articles were selected for all matrices using Medline and ISI Web of Knowledge by reading the titles and abstracts. Only studies that met the inclusion criteria were selected and read in full. Google Scholar was used for additional search. After careful consideration, a total of 52 articles were included in this review.

## 3. Cannabinoid Determination in Conventional Biological Samples

The next lines describe the determination of cannabinoids in the most commonly used biological samples, namely blood (and derivatives) and urine.

### 3.1. Whole Blood, Plasma, and Serum

Blood offers precious information regarding recent drug use, and its analysis allows assessing the degree of influence [36]. In general, this specimen allows correlating drug levels to the observed symptoms or to the degree of impairment. However, concentrations of the drugs are usually low, and this is particularly true for cannabinoids, being expected concentrations in the low ng/mL range. In addition, sample collection must be performed by specialized personnel, as it is extremely invasive and onerous to the donor.

In this sample, recent exposure to cannabis is monitored through the analysis of THC, THC-OH, and THC-COOH [2]. THC can be found in blood samples collected right after first inhalation, since TCH levels peak after just 8 min of exposure. Interestingly, concentration ratios of THC and metabolites can indicate the consumption timeline. A THC:THC-OH ratio of 2:1 is present 2 to 3 h after consumption [37], for example. The ratio between THC-COOH-glucuronide (THC-COOH-gluc) and THC-COOH varies from around 0.5 to 5. This variation depends on frequency and time since consumption. The Swiss Society of Legal Medicine (SGRM) has proposed a cut-off value of 40 ng/mL for THC-COOH [38].

THC, THC-OH, and THC-COOH are frequently found in plasma [39], and THC concentrations of over 3 ng/mL are recognised as a sign of recent ingestion. In addition, plasma analysis can determine the degree of CBD exposure [37].

For anti-doping analysis, THC-COOH screening in serum is usually required [37].

Analytical methods developed in the last three years for whole blood, plasma, and serum samples are displayed in Table 1.

Even though there are several reported procedures for the analysis of cannabinoids in these matrices using GC-MS methodologies, in general, blood and plasma are difficult to analyze by GC due to matrix interferences [49]. There are low volatility components in these matrices that can obstruct the GC active sites and its injection liner or column, leading to the degradation of the analytes or an irreversible adsorption in those active sites. The result is an increased signal of the analyte in this case when compared to cases in which there are no interferents, which can overestimate cannabinoids’ concentrations. There are some reported solutions to deal with this, but they demand a laborious sample treatment process [50] and modifications to the equipment [51], can easily lead to column deterioration, and present limited effectiveness [52].

The matrix effect in GC-MS regarding the analysis of cannabinoids is mitigated in the recent work of Dawidowicz et al. [40]. The authors added oleamide to the sample extracted via a Quick, Easy, Cheap, Effective, Rugged and Safe (QuEChERS) methodology and reached values of limits of detection (LOD) exhibiting higher sensitivity and less matrix effects than most authors. The analyte response signal increased significantly, but there was also a risk of decreased analyte quantity due to polymerization of THC and its metabolites by metal ions present in blood. To minimize the risks and increase efficacy, the authors advocate that the addition of oleamide should be performed just before injection and that the magnitude of the signal depends on the ratio of the analyte to oleamide. The frequently used dilute and shot analysis for biological specimens may present some drawbacks in what concerns cannabinoid determination, since ion suppression caused by matrix interferences may hinder chromatographic peaks. Sørensen and Hasselstrøm [53] have proposed an interesting approach to overcome this problem, namely a quick filtration step to efficiently remove co-eluting phospholipids before LC-MS/MS analysis of whole blood extracts.

Products containing Δ^8^-THC have become more prevalent over the years. This isomer has been identified by GC-MS, but it is common to obtain interfering peaks for Δ^8^-THC and 11-nor-9-carboxy-Δ^8^-tetrahydrocannabinol (Δ^8^-THC-COOH) isomers [8]. Considering their structural resemblance (as seen in Figure 1), it is extremely difficult to accurately quantify Δ^8^ and Δ^9^ isomers in toxicological analysis [41].

The team of Chan-Hosokawa [8] noticed several cases of overlapping peaks in cannabinoid confirmation tests using LC-MS/MS. These cases cannot be considered since they do not meet the acceptance criteria and can lead to false positive or inconclusive results [41]. Thus, the group developed an analytical methodology to separate Δ^8^ and Δ^9^ isomers and their metabolites in blood samples. Extending the run time of analysis seemed to solve this problem, but since this is impractical for routine tests, the team decided to maintain its original run time and reanalyze cases with overlapping peaks with the longer run-time method. However, to avoid evaporation of the samples, vial tops should be replaced after the first analysis, or reinjection should take place within 24 h [8].

Likewise, Reber et al. [41] developed an analytical methodology to quantify Δ^8^-THC, Δ^9^-THC, Δ^8^-THC-COOH, and Δ^9^-THC-COOH in blood and urine samples with a SPE-LC-MS/MS methodology. While Δ^8^-THC and Δ^9^-THC results were satisfactory, a deuterated form of ∆^8^-THC-COOH would help increase accuracy values for the study of the carboxylated isomers. The team applied this analysis to real samples that would have been analysed with no monitorization of the Δ^8^ isomers and found that in some cases ∆^9^ cannabinoid concentrations were below established LODs, and as such would have been reported as negative.

In 2020, Hubbard et al. [44] developed an LC-MS/MS analytical methodology to quantify THC, tetrahydrocannabivarin (THCV), THC-OH, THC-COOH, THC-COOH-gluc, CBN, CBD, and cannabigerol (CBG) in whole blood samples. Extraction was performed with SPE, and the lower limits of quantification (LLOQ) ranged from 0.5 to 2 ng/mL. This method was applied by the same team one year later to study a biomarker that suggests recent cannabis consumption [54]. They learnt if it was possible to identify and quantify cannabinoids in blood, OF, or breath in the period of greatest impairment—the first 3 h after consumption. Samples were collected before and 6 h after smoking. THC, THC-COOH, and THC-COOH-gluc were identified in most samples collected after consumption. THCV and CBD were rarely detected. The team was able to conclude that THC’s concentration in cannabis is not directly related to the resulting THC concentration in blood since factors such as puff volume and puff duration may influence this value. It was concluded that CBN is the best suited cannabinoid to be used as a biomarker for recent use. This analyte—which is a primary degradation product of THC—showed the best results at the cut-off value. CBN’s concentration drops faster than THC’s, being less likely to be identified 3 h after consumption. In chronic users THC-OH, THC-COOH, and THC-COOH-gluc can be detected in blood days to several weeks after consumption, so these analytes are not suited to being biomarkers for recent use. The same happens with CBD (since its concentration depends on cannabis preparations and can be present in formulations with no THC), CBG, THCV, THC-glucuronide (THC-gluc), and THCA-A (which are not frequently detected in either blood or OF samples).

The team of da Silva et al. [39] applied for the first time a salting-out assisted liquid-liquid extraction (SALLE) and LC-MS/MS methodology to analyse THC, THC-OH, THC-COOH, CBN, and CBD in plasma. The salting-out effect of SALLE separates a water-miscible organic solvent from plasma, which induces protein precipitation and separation of the phases. This extraction technique is able to extract compounds with different polarities, which is the case of different cannabinoids.

Pichini et al. [47] validated an ultra-high-performance liquid chromatography-tandem mass spectrometry (UHPLC-MS/MS) methodology to analyse THC, THC-gluc, THCA-A, THC-OH, THC-COOH, THC-COOH-gluc, CBD, and CBDA in plasma, urine, OF, and sweat samples of individuals treated with medicinal cannabis. The team developed a dilute and shoot procedure, hence avoiding time-consuming extraction methodologies. An UHPLC-MS/MS methodology operating in positive electrospray (ESI) mode allowed the team to be able to detect minimal quantities of the cannabinoids studied. The method was successfully applied to real samples.

### 3.2. Urine

Urine analysis has been used extensively in workplace drug testing and in abstinence control programs, as it gives important information on recent exposure [36,55,56]. However, this does not apply for cannabis [57], as several factors such as frequency of use, timing of sample collection, body fat, and urine dilution contribute to the detectability of THC metabolites in urine [37]. Samples such as OF or exhaled breath are better suited for this purpose.

This matrix is reliable and easy to collect, and its analysis is inexpensive. Thus, it is the most frequently published matrix in cannabinoids’ analysis [37]. However, one should consider the infringement of the donor’s privacy in controlled sampling, and the real possibility of sample adulteration or substitution in uncontrolled settings.

Little to no THC or THC-OH can be found in urine, so THC-COOH is the most suited cannabinoid to prove consumption. Indeed, for anti-doping analysis, THC-COOH screening in urine is usually required, as 20% of cannabis is excreted in this sample as THC-COOH and THC-COOH-gluc [37]. The THC-COOH-gluc:THC-COOH ratio varies from around 1.3 to 4.5, so monitorization of THC-COOH-gluc is often deemed necessary [38]. Another possibility of detecting the metabolites is via sample alkaline digestion [58].

Cut-off values for cannabinoids in urine samples have been proposed by the Substance Abuse and Mental Health Services Administration (SAMHSA) [59] and by the European Workplace Drug Testing Society (EWDTS) [60], as summarized in Table 2. There are no proposed cut-off values for CBN and CBD.

According to SAMHSA, a cut-off value of 50 ng/mL is accepted for THC-COOH screening and a 15 ng/mL value must be reached for confirmation analysis [59]—the EWDTS also reports this value [60].

Analytical methods developed in the last three years for urine samples are displayed in Table 3.

Rosendo et al. [58] were the first team to extract THC, THC-OH, THC-COOH, CBN, and CBD from urine samples using a microextraction by packed sorbent (MEPS) technique to pre-concentrate the compounds, which were later analysed by GC-MS. The method was applied successfully to authentic samples and proved efficient. As mentioned before, modern microextraction techniques are cheaper, faster, require fewer amounts of sample and organic solvents, and have good extraction efficiencies.

Likewise, Morisue Sartore et al. [63] developed a new packed-in-tube solid-phase microextraction (IT-SPME) technique coupled to LC-MS/MS to automatically extract THC, THCV, THC-OH, THC-COOH, THC-COOH-gluc, CBN, and CBD from urine samples. Thus, both metabolites and neutral cannabinoids were analysed. The microcolumn for the packed IT-SPME-LC-MS/MS was extremely robust, being reused over 150 times, which dismisses the use of commercial devices. The method was applied successfully to authentic samples and proved efficient.

Urine is also a well-known specimen for the estimation of CBD exposure [37,64]. Ameline et al. [64] developed a GC-MS/MS methodology to detect CBD in urine, OF, hair, exhaled breath, and sweat after administration of a CBD capsule to a human volunteer, filling a gap when alternative matrices are concerned. Since the team expected low concentrations of the analyte, they decided not to use the correspondent deuterated compound. With the analysis of these five different matrices, the team managed to extend the detection window of CBD from 48 h (in urine) to 144 h (in sweat). Concerning urine, non-hydrolyzed samples tested negative, but enzymatic hydrolysis provided positive results, which proved that this step is vital to analyze CBD-gluc, the excreted form of CBD. Other authors share this conclusion [65,66].

**Table 3:** Methods for the identification and quantification of cannabinoids in urine.

Amount (µL)	Analyte(s)	Extraction (Extraction Solvent)	Derivatization	Detection Technique (Acquisition Mode)	Linearity (ng/mL) LOD and LOQ (ng/mL) Injection Volume (μL)	Reference
1000	THC, Δ^8^-THC, THC-COOH, and Δ^8^-THC-COOH	SPE [hexane/ethyl acetate/glacial acetic acid (49:49:2, v/v/v)]	N/A	LC-MS/MS (MRM-ESI+)	Linearity: THC and Δ^8^-THC: 1 to 50; THC-COOH and Δ^8^-THC-COOH: 5 to 250 LOD: THC and Δ^8^-THC: 1; THC-COOH and Δ^8^-THC-COOH: 5 LOQ: THC and Δ^8^-THC: 1; THC-COOH and Δ^8^-THC-COOH: 5 Injection volume: 10	Reber et al., 2022 [41]
250	THC, THC-OH, THC-COOH, CBN, and CBD	SPE (acetonitrile)	MSTFA	GC-MS/MS (SRM-EI)	Linearity: THC: 0.3 to 20; THC-OH: 0.3 to 15; THC-COOH: 3 to 150; CBN: 0.2 to 12; CBD: 0.3 to 20 LOD: THC, THC-OH, and CBD: 0.15; THC-COOH: 1; CBN: 0.1 LOQ: THC, THC-OH, and CBD: 0.3; THC-COOH: 3; CBN: 0.2 Injection volume: 1	Frei et al., 2022 [38]
500	THC and THC-COOH	QuEChERS [acetonitrile; H_2_O; anhydrous MgSO_4_/NaOAc (4:1); primary and secondary amine and MgSO_4_]	N/A	UHPLC-MS/MS (MRM-ESI+)	Linearity: THC: 4 to 400; THC-COOH: 10 to 240 LOD: THC: 1; THC-COOH: 4 LOQ: THC: 4; THC-COOH: 10 Injection volume: 1	Ferrari et al., 2022 [42]
250	THC, THC-OH, THC-COOH, CBN, and CBD	MEPS (C_8_ and SCX) [methanol and water, washing with 0.1% formic acid in water with 5% isopropanol; elution 0.1% ammonium hydroxide in methanol]	MSTFA with 5% TMCS	GC-MS (SIM-EI+)	Linearity: THC and CBD: 1 to 400; THC-OH and CBN: 5 to 400; THC-COOH: 10 to 400 LOD: THC, THC-OH, and CBD: 1; THC-COOH and CBN: 5 LOQ: THC and CBD: 1; THC-OH and CBN: 5 THC-COOH: 10 Injection volume: 3	Rosendo et al., 2022 [58]
250	THC, THCV, THC-OH, THC-COOH, THC-COOH-gluc, CBN, and CBD	IT-SPME (acetonitrile)	N/A	LC-MS/MS (MRM-ESI+)	Linearity: THC, THCV, THC-OH, THC-COOH, CBN, and CBD: 10 to 160; THC-COOH-gluc: 25 to 1000 LOD: N/A LOQ: THC, THCV, THC-OH, THC-COOH, CBN, and CBD: 10; THC-COOH-gluc: 25 Injection volume: N/A	Morisue Sartore et al., 2022 [63]
N/A	THC-COOH	DLLME [acetonitrile (disperser solvent) and chloroform]	BSTFA with 1%TMCS	GC-MS/MS (MRM-EI+)	Linearity: 5 to 500 LOD: 1 LOQ: 5 Injection volume: 2	Rodrigues et al., 2022 [67]
2000	TCH-COOH and CBD	LLE (*tert*-butyl-methyl ether)	MSTFA/NH_4_I/ethanethiol (1000/ 2/3; v/w/v)	GC-MS/MS (SRM-EI)	Linearity: 5 to 50 LOD: THC-COOH: 3.7; CBD: 5.1 LOQ: N/A Injection volume: 2	Danila et al., 2022 [68]
10	THC-COOH	Biofluid/methanol (70:30, v/v)	Fast Red RC derivatization reagent	PS-MS/MS (SRM-ESI+)	Linearity: 2 to 250 LOD: 1.3 LOQ: 10 Injection volume: N/A	Borden et al., 2022 [69]
500	THC-COOH	LLE [methanol/acetonitrile (80:20, v/v)]	N/A	UHPLC-MS/MS (SRM-ESI-)	Linearity: 10 to 250 LOD: 3 LOQ: 6 Injection volume: 4	Gerace et al., 2021 [70]
1000	CBD	LLE [hexane/ethyl acetate (90:10, v/v)]	BSTFA + 1% TMCS	GC-MS/MS (MRM-EI)	Linearity: 0.01 to 100 LOD: 10 LOQ: N/A Injection volume: 1	Ameline et al., 2020 [64]
100	THC, THC-gluc, THCA-A, THC-OH, THC-COOH, THC-COOH-gluc, CBD, and CBDA	LLE [acetone:acetonitrile (80:20, v/v)]	N/A	UHPLC-MS/MS (MRM-ESI+)	Linearity: N/A LOD: THC: 0.04; THC-gluc: 0.07; THCA-A and THC-OH: 0.06; THC-COOH: 0.08; THC-COOH-gluc: 0.09; CBD: 0.05; CBDA: 0.065 LOQ: THC: 0.09; THC-gluc: 0.14; THCA-A and THC-OH: 0.11; THC-COOH: 0.18; THC-COOH-gluc: 0.19; CBD: 0.1; CBDA: 0.12 Injection volume: 10	Pichini et al., 2020 [47]
15,000	THC, THC-OH, and THC-COOH	Automated MEPS (90% acetonitrile)	N/A	LC-MS/MS (MRM-ESI+)	Linearity: THC and THC-OH: 25 to 250; THC-COOH: 5 to 170 LOD: THC and THC-OH: 5; THC-COOH: 1 LOQ: THC and THC-OH: 20; THC-COOH: 5 Injection volume: N/A	Sartore et al., 2020 [71]

Legend: BSTFA [*N*,*O*-Bis(trimethylsilyl)trifluoroacetamide)]; CBD (Cannabidiol); CBDA (Cannabidiolic acid); CBN (Cannabinol); DLLME (Dispersive liquid-liquid microextraction); EI (Electron ionization); ESI (Electrospray ionization); GC-MS/MS (Gas chromatography-tandem mass spectrometry); IT-SPME (In tube solid-phase microextraction); LC-MS/MS (Liquid chromatography-tandem mass spectrometry); LLE (Liquid-liquid extraction); LOD (Limit of detection); LOQ (Limit of quantification); MEPS (Microextraction by packed sorbent); MRM (Multiple reaction monitoring); MSTFA (*N*-Methyl-*N*-trimethylsilyl-trifluoroacetamide); N/A (Not available); PS-MS/MS (Paper spray-tandem mass spectrometry); QuEChERS (Quick, Easy, Cheap, Effective, Rugged and Safe); SPE (Solid-phase extraction); SIM (Selected Ion Monitoring); SRM (Selected reaction monitoring); THC (Δ^9^-tetrahydrocannabinol); THCA-A (Δ^9^-tetrahydrocannabinolic acid A); THC-COOH (11-nor-9-carboxy-Δ^9^-tetrahydrocannabinol); THC-COOH-gluc (11-nor-9-carboxy-Δ^9^-tetrahydrocannabinol-glucoronide); THC-gluc (Δ^9^-tetrahydrocannabinol-glucoronide); THC-OH (1-hydroxy-Δ^9^-tetrahydrocannabinol); THCV (Tetrahydrocannabivarin); TMCS (Trimethylchlorosilane); UHPLC-MS/MS (Ultra-high-performance liquid chromatography-tandem mass spectrometry); Δ^8^-THC (Δ^8^-tetrahydrocannabinol); Δ^8^-THC-COOH (11-nor-9-carboxy-Δ^8^-tetrahydrocannabinol).

## 4. Cannabinoid Determination in Unconventional Biological Samples

A great deal of attention is being paid to less-used matrices for drug testing, the so-called alternative or unconventional samples. This is because of the advantages they present when compared to conventional specimens, as will be further discussed below.

### 4.1. Oral Fluid/Saliva

OF comprises saliva (secretions from the salivary glands) and other products that can be found in the oral cavity [11]. This sample has been used in the medical field to help diagnose oral and systemic disease markers and to monitor drugs and hormones [72]. Nowadays, OF analysis is used in cases of drug treatment, workplace drug testing, pain management, and DUID programs [11].

The advantages of OF testing include the possibility of on-site collection and screening, and the fact that OF is more likely to contain parent drugs (which may reflect recent drug use) [11]. The collection procedure is the main advantage for its use, since it is not only non-invasive and moderately easy to perform by non-medical personnel, but it can also be achieved under supervision to prevent adulteration or substitution of the samples [72]. Furthermore, there is a low biohazard risk during collection, and it is possible to collect multiple samples [11].

There are several disadvantages of OF testing, such as the lack of sample available for analysis—that can be caused either by physiological aspects or because of the drug itself [36,73]. THC, particularly, causes “dry mouth” [74]. Food, drinks, mouthwash, or anti-THC spray influence THC concentrations in OF samples but do not mask recent consumption [75]. There are also stimulation techniques that can cause an incorrect quantification of the drug [73].

This biological sample can be collected by several different techniques, such as passive drool or stimulation of expectoration and saliva. Passive drool is the method that best reflects drug concentrations but is slow and unpleasant for both donors and collectors. Expectoration or spitting are also disagreeable for both parties and may contain several interferences but provide great sensitivity. These methods do not use a stabilizing buffer, which may lead to lower drug stability—which, in fact, happens with cannabinoids [11].

Several collection devices can also be employed for sample collection [11]. These devices usually contain a pad to absorb the sample and a buffer to stabilize the drugs. The pads filter the samples (which reduces the interferences), and the buffers reduce viscosity (which improves measurement accuracy and stability but also dilutes drug concentrations). Especially in cannabis testing, it is important to allow sufficient time for the pad to interact with the buffer to obtain maximal drug recovery [62].

Various authors state that this matrix is the only body fluid in which drug levels would correlate to those in blood [1]. However, THC can be detected in OF samples immediately after consumption, but this analysis concerns the THC present in the mouth, not the concentration present in the blood at that moment [75]. Rinsing the mouth with water would actually significantly reduce the THC concentration [11]. In the first few hours after consumption, the concentration of THC in OF is generally higher than the concentration of the analyte in blood [75]. The THC present in OF is in fact mostly from oral mucosa contamination, as opposed to transferred from blood [11]. In addition, THC will persist in blood longer than in OF [75]. Thus, it is not possible to predict the THC concentration in blood by studying the concentration in OF, even though the evolution of this analyte’s concentration in these matrices has similar tendencies [11,75].

THC concentrations detected in OF range from 1 to several thousand ng/mL, so they cannot be translated in terms of behavioural changes. The concentrations of THC in this matrix drop quickly, reaching values lower than 5–10 ng/mL two to four hours after smoking [54,75]. Still, in cases of chronic users, this analyte can maintain values of 1–5 ng/mL for more than forty-eight hours [75]. Recent cannabis use can be confirmed if THC concentrations in OF are above 10 ng/mL [20,54,75,76]. This concentration value is typical of a period of altered consciousness and behaviour [20,75].

According to SAMHSA, the cut-off value for THC screening is 4 ng/mL, and a value of 2 ng/mL is admitted for confirmation [61]. The EWDTS proposes a cut-off value for THC screening of 10 ng/mL [62]. These values are summarized in Table 2.

In occasional users (either by smoked, vaporized, or oral consumption), the cut-off value for THC can still be reached 26 h after exposure. In frequent users, the same happens over 72 h later. Thus, similarly to blood and urine, OF analysis can detect low THC concentrations for several days. In frequent and occasional users, CBG, CBN, and THCV have also been reported 26 h after exposure [11].

Parent drugs are more prevalent in this matrix than their metabolite, but THC-COOH monitorization provides additional information. This metabolite is rarely detected in OF samples, and can be present in blood, or from the metabolism of THC in the oral mucosa [11,77]. Chronic users showcase positive results, although at very low concentrations, usually lower than 0.05 ng/mL [20,75]. It is possible to increase THC-COOH detectability in OF samples if they undergo hydrolysis prior to analysis. Since this metabolite is not present in cannabis smoke, it can be monitored to confirm cannabis consumption. Its identification proves Marinol^®^ intake, for example, which THC does not [11].

There is no established marker impairment for cannabis consumption, so in DIUD cases a zero to 5 ng/mL tolerance is usually applied for blood samples [2,54]. This is not a good practice since some cannabinoids can be detected in chronic users’ blood over 30 days after the last consumption. Another indicative that blood is not the best matrix to collect in DIUD cases is the 1.5 h timeframe that usually occurs between a traffic stop and blood draw. In this period, the concentration of THC may drop up to 90% [2], but “high” can persist for several hours depending on the user [54]. Thus, alternative samples such as OF should be considered [2].

So far, no biomarkers in OF samples correlate well with the pharmacodynamic effects of THC in the body [20]. Nevertheless, CBN and CBD are more related to the duration of cannabis effects [54,76]. The presence of these analytes in OF samples varies greatly depending on the type of cannabis consumed. CBN and CBD have shorter detection windows than THC in this matrix [75].

When analysing the results from OF samples, it is important to note different factors that may influence a drug’s detectability, such as their route of administration and individual characteristics [11].

When it comes to CBD-based products, only THC concentrations higher than 25 ng/mL in OF samples indicate that it is not a case of merely passive contamination or exposure. A behaviour change would also solidify this statement [75].

Some studies have been conducted to test if passive smokers produce positive samples for cannabinoids after exposure. In a 2004 study conducted by Niedbala et al., the team determined that positive results after passive cannabis smoke inhalation are limited to around thirty minutes after exposure [78]. Other studies state that passive exposure to cannabis may provide positive THC results in OF samples, although concentrations are usually low (<5 ng/mL), and the effect can dissipate within three hours. Higher concentration values are expected if exposure was in non-ventilated rooms. Even collection devices can be contaminated [11].

OF samples can be analysed with the same analytical methods and sample volumes as blood and urine. However, the volumes required for testing depend on several factors, such as the type of sample collection, the amount of analytes, and the sensitivity of the analytical method [11].

Lin et al. [79] developed a sensitive and specific LC-MS/MS methodology for the quantification of ∆^8^-THC, THC, THC-OH, THC-COOH, CBN, CBD, cannabidiorcol (CBD-C1), CBG, cannabichromene (CBC), CBDA, cannabidivarin (CBDV), THCA-A, and THCV in OF samples. The team was the first to develop an analytical methodology that would monitor ∆^8^-THC alongside other cannabinoids in OF. They obtained lower limits of quantification (LOQ) for THC, CBD, THC-OH, THC-COOH, CBN, THCV, and CBG when compared with other published methods. The developed methodology was applied to real samples, and all analytes were confirmed in at least one sample. They found high concentrations of over 400 ng/mL of THC-OH and THC-COOH in three samples, which is not common.

There are some considerations in the analysis of cannabinoids in this sample. In 2012, Andrews and Paterson [80] indicated a potential conversion of CBD to THC/∆^8^-THC in GC-MS analysis when acidic derivatives were used. In 2019, the National Laboratory Certification Program (NLCP) [81] stated a potential analytical conversion of CBD to THC-COOH in urine samples (also using GC-MS and acidic derivatization agents). A year later, Golombek et al. [82] concluded that there was in fact a conversion from CBD to THC, but these conversions did not occur in vivo. Likewise, in OF samples CBD can convert to THC and ∆^8^-THC in strong acidic conditions. Thus, controlling this analytical conversion is extremely important to understand the nature of consumption, since THC findings may not necessarily indicate THC ingestion. Since CBD is widely used in non-psychotropic products, this understanding can differentiate between consumption patterns. Considering this conversion, if both THC and CBD are present in a sample, there could be an over- or under-reporting situation for both cannabinoids. In 2021, Coulter et al. [83] studied the analytical conditions that let CBD convert to THC by comparing different sample preparation and extraction techniques. The team developed an analytical methodology to circumvent this problem based on a SPE with Cerex Polycrom THC and LC-MS/MS methodology. This method, however, did not find THC-COOH at lower concentrations.

Another interesting method to collect OF samples is the use of dried oral fluid spots (DOFS). Gorziza et al. [84] developed for the first time a LC-MS/MS methodology to extract THC and CBD from DOFS samples. Briefly, a small quantity of liquid OF is applied to a paper substrate that air dries. This method improves xenobiotics’ stability when compared to liquid samples, which would be an advantage if used in DIUD cases since it would facilitate transportation and storage. However, the team did not achieve good recovery values.

The work previously mentioned by Ameline et al. [64] was the first to dosage CBD in this matrix after oral consumption of pure CBD. Since the detection of cannabinoids in OF is mainly due to contamination of the oral cavity—which does not happen when a capsule is consumed—the interpretation of the results was difficult.

Spindle et al. [20] compared cannabinoid concentrations in whole blood and OF samples after administration via smoking and vaporization. OF analyses did not follow the same time course as whole blood, thus concluding that residual deposition of THC in the oral cavity may influence the results. THC concentrations in OF peaked within 10 min for both inhalation methods and declined rapidly thereafter. The authors have further concluded that pharmacokinetics varied according to the inhalation method and the tested biological specimen. Vaporization appeared as a more efficient way of compound delivery when compared with smoking.

Analytical methods developed in the last three years for OF samples are displayed in Table 4.

### 4.2. Hair

Hair analysis has been increasingly studied in the last few years. Considering that most abusive and therapeutic drugs can be detected in the hair of chronic users, this specimen has been of utmost importance in the field of forensic and clinical toxicology, providing valuable information concerning the history of abuse.

Xenobiotics are absorbed into growing cells in the hair follicle by passive diffusion from the blood. They remain trapped in the keratinous matrix without further metabolism [28], so hair analysis provides a window of detection that can extend to several months (or even years; as long as the length of the hair allows) [1,2]. Thus, hair analysis gives information about chronic use or a single exposure [29]. Furthermore, drugs are usually stable in hair, and hair is a strong and stable tissue, which are examples of the advantages this sample has over conventional biological matrices such as blood or urine [2,29,88]. Moreover, hair is less affected by adulterants or short-term abstinence, can be stored for long periods of time, does not require refrigeration, and adulteration is difficult [28,29].

Sample collection is simple and non-invasive. The preferred area to collect the samples is the posterior vertex region of the head, close to the scalp, since it has the least variation in growth rates. A pencil thickness of hair is sufficient to perform routine and confirmation tests [88].

All these advantages lead to hair analysis being used in cases of revocation of driving licenses, alleged drug addiction, follow-up of detoxication treatments, monitoring of withdrawal, child protection, and workplace drug testing, being already routinely used in the fields of forensic and clinical toxicology and traffic and occupational medicine [2,29]. Hair analysis is particularly important in cases of drug-facilitated crimes, since the substances that are commonly associated with these occurrences cause sedation and amnesia, so the victims may report the crimes only after a considerable amount of time [29].

The main disadvantage of hair analysis is the fact that there is still a lack of information regarding drugs’ incorporation mechanisms in hair, which makes interpreting results challenging [2]. In addition, cosmetic treatments may alter drug concentrations in hair to different extents, depending on which treatment was used [29,88]. Individual characteristics such as ethnicity may too be relevant; black or brown hair tends to accumulate more basic-type drugs, due to higher levels of eumelanin, for example. Furthermore, there has been reported contamination of THCA after manipulation of cannabis material and contact with side stream smoke. All these factors must be taken in consideration when evaluating the obtained data. Colour, length, site of collection, and cosmetic treatments should be recorded when the sample is collected [1,29].

It is therefore of upmost importance to wash hair samples prior to analysis, to remove external contaminants [29] and any of the analytes adsorbed on the hair surface [6]. This procedure should include both organic solvents and aqueous solutions, since the first will remove only surface contamination, and the latter will swell the hair and extract drugs from within the matrix [29]. Methanol is also widely chosen as a washing solvent because it provides efficient solubilization of contaminants and keeps the integrity of the matrix [28]. Deuterated water and dichloromethane have also been reported [6].

This pre-treatment step is followed by digestion of the hair, which allows the release of the analytes from the matrix [89]. Alkaline hydrolysis is usually employed [3,6]. It is noteworthy to point out that THC can be formed from decarboxylation of THCA after hair digestion at elevated temperatures, so this procedure should be performed with caution.

The resulting extract can then be analysed by screening techniques or undergo further clean-up procedures.

To help differentiate between contamination and ingestion, confirmation methods should determine the parent drug and its metabolites [29]. To study cannabis exposure in hair, THC, THC-COOH, CBN, and CBD are usually investigated [89]. According to the Society of Hair Testing (SoHT), the cut-off values to prove cannabinoid ingestion are 50 pg/mg (0.05 ng/mg) for THC and 0.2 pg/mg (0.0002 ng/mg) for THC-COOH. The cut-off value for THC screening is 100 pg/mg (0.1 ng/mg) [29]. These values are established in spite of the fact that THC is the main cannabinoid accumulating in hair, and identification and quantitation of compounds present in cannabis smoke (like THC, THCA-A, CBN, and CBD [3]) are not sufficient to prove cannabis intake, since it does not dismiss passive exposure—hair can become contaminated with THC through smoke, dust, or even dirty hands. Thus, the metabolite THC-COOH should be monitored as well, since this compound is formed exclusively within the body [3,28]. These values are summarized in Table 2.

However, the main analytical challenge when it comes to hair testing for cannabis intake is the identification and quantification of THC-COOH. The acidic nature of THC-COOH leads to its critically low concentration levels in hair due to the preferential incorporation of basic compounds into the hair shaft [7]. In addition, there are several other aspects that can contribute to further complications in this analysis: the low weight of the hair sample, the fact that hair contains structurally similar lipophilic organic acids, the frequency of consumption, and genetic factors [6,90]. Thus, this metabolite is usually present in extremely low concentrations in this matrix, so its detection may be difficult because of the background interference from the hair matrix. Highly sensitive analytical techniques are therefore required to detect it [3], since the cut-off value recommended by the SoHT is not reachable with a single mass spectrometer [89]. It is also noteworthy to point out that because THC-COOH is hydrophobic, it tends to adsorb on containers, pipette tips, and/or filtration devices, so high volumes of organic solvents are needed to circumvent this problem [91].

On the other hand, a cut-off value for THC-OH has not been fixed by the SoHT. Casati et al. [7] proposed a value of 0.5 pg/mg for this analyte in both scalp and body hair to differentiate between chronic use and external contamination and suggested that the detection of both THC-COOH and THC-OH should be required to prove active intake of cannabis. Hair incorporation of neutral cannabinoids such as THC and THC-OH is higher than that of acidic metabolites such as THC-COOH. Consequently, THC-OH is expected to have higher concentration in hair than THC-COOH, since the first is less polar than the latter, and drug lipophilicity is directly correlated with the extent of hair deposition. Thus, the team showed that monitoring THC-OH alongside THC-COOH could improve detection of cannabis consumption and avoid false results. Other authors have agreed with this statement [6].

Casati et al. [7] also found that the levels of THC-OH and THC-COOH found in body hair were higher than those found in scalp hair, which can be explained by less exposure to external conditions and cosmetic treatments, differences in pigmentation, and an increased incorporation from sweat or sebum.

According to the guidelines of the SoHT, for the diagnosis of active consumption of these substances, GC-MS/MS is the required method, considering that the recommended cut-off value for THC-COOH is unreachable when using a single mass spectrometer. Indeed, most of the methods that have already been proposed for the determination of these compounds in hair are based on GC and require derivatization of the sample to identify THC-COOH. However, there has been an increase in the development of LC-MS/MS methodologies for the identification and quantification of cannabinoids in hair, but the number of articles reporting the use of LC-MS/MS for the identification and quantification of THC-COOH in hair is still low [3,28].

Analytical methods developed in the last three years for hair samples are displayed in Table 5.

Although LC-MS/MS techniques do not usually require derivatization, the fact is that most developed methodologies for THC-COOH do not obtain the desired cut-off value [6]. In 2021, Al-Zahrani et al. [6] developed and validated a LC-MS/MS methodology to detect THC, THC-COOH, and CBN in hair. The analytes were extracted from the hair by polymeric strong anion mixed mode SPE. Before injection into the chromatographic system, the team performed a derivatization procedure, which is not common when employing a LC-MS/MS methodology. Methanolic HCl and 2-fluoro-1-methylpyridinium-*p*-toluenesulfonate (FMP-TS) were added to the samples to derivatize carboxyl and phenolic groups, respectively. The step allowed the formation of a methyl ester, which increased the compound’s lipophilicity and removed the negative charge on the carboxyl group, consequently improving LOD values. For THC-COOH, the LOD was 0.1 pg/mg, and the LOQ was 0.2 pg/mg, which is in accordance with the values proposed by the SoHT.

Body hair has also been studied. In the work previously mentioned by Ameline et al. [64], the team was the first to analyze beard hair to study CBD, 7 and 14 days after oral ingestion of a capsule. CBD has a weak affinity to melanin, which has better affinity to alkaline substances, especially if they have a nitrogen atom. This fact reduces the detection rates of this cannabinoid in this matrix, hence the lower concentrations the team found in this study. Furthermore, it is noteworthy to point out that xenobiotics’ incorporation in beard hair is different than that of scalp hair considering the differences in growth rate and cycle.

In 2019, Cho et al. [3] developed a method to identify and quantify THC-COOH in the hair of drug abusers using LC-MS/MS. THC-COOH was extracted by LLE in acidic conditions and analysed in a LC-MS/MS system in multiple reaction monitoring (MRM) with ESI-mode for identification and quantitative analysis. Then, a triple stage mass spectrometry (MS^3^) analysis was performed for reconfirmation of THC-COOH detection. The team used a previously reported [92] column-switching valve system to remove the high background level of the matrix lipids that are produced from the alkaline hydrolysis of the hair. This column-switching procedure removes matrix interference, providing sufficient selectivity for drugs in complex biological specimens such as hair. This system consisted of a pre-column, a trap column, and an analytical column. The combination of this technique with the use of both MRM and MS^3^ acquisition modes made it possible for the team to achieve LOD and LOQ values of 0.1 pg/mg. The team reached the cut-off value proposed by the SoHT for THC-COOH but did not reach the cut-off value of 0.05 pg/mg recommended by Korean forensic labs. The use of both acquisition modes also increased the method’s reliability [3].

In 2014, Dulaurent et al. [89] developed a method to simultaneously identify and quantify THC, THC-COOH, CBN, and CBD in hair using LC-MS/MS. The team used a mixture of pentan-1-ol/methanol with 0.1% formic acid/pure water with 0.1% formic acid (50:30:20, v/v/v) to reconstitute the dried residues after evaporation to dryness. Pentan-1-ol was used after they noticed that at the dry residue step, the analytes were trapped by oily drops that their LC-MS/MS injection phase could not solubilize. Adding pentan-1-ol solved the problem considering its both polar and apolar behaviour (for an affinity with the injection phase and an affinity with the lipids from the hair, respectively). This, alongside the use of MS^3^ mode, made it possible to reach the cut-off value for THC-COOH.

**Table 5:** Methods for the identification and quantification of cannabinoids in hair.

Sample	Amount (mg)	Analyte(s)	Washing	Digestion	Extraction (Extraction Solvent)	Derivatization	Detection Technique (Acquisition Mode)	Linearity (ng/mL) LOD and LOQ (ng/mL) Injection Volume (μL)	Reference
Scalp hair	20	THC-OH and THC-COOH	Isohexane and acetone	1 M NaOH, 80 °C, 1 h	LLE [isohexane/ethyl acetate mixture (90:10, v/v)]	THC-OH: Picolinic acid	LC-MS^3^ (MRM-ESI-)	Linearity: 0.1 to 15.0 LOD: THC-COOH: 0.08 LOQ: THC-COOH: 0.1 Injection volume: 20	Hehet et al., 2022 [93]
Scalp hair	20	THC, THC-OH, THC-COOH, CBD, 6-α-OH-CBD, 6-β-OH-CBD, 7-OH-CBD, and 7-COOH-CBD	Dichloromethane (thrice)	M3^®^ reagent, 100 °C, 1 h	N/A	N/A	THC: UHPLC-MS/MS (MRM-ESI+) THC-OH, THC-COOH, CBD, 6-α-OH-CBD, 6-β-OH-CBD, 7-OH-CBD and 7-COOH-CBD: UHPLC-MS/MS (MRM-ESI-)	Linearity: THC, THC-OH, CBD, 7-OH-CBD, and 7-COOH-CBD: 50 to 5000; THC-COOH, 6-α-OH-CBD, and 6-β-OH-CBD: 0.2 to 1000 LOD: THC, THC-OH, CBD, 7-COOH-CBD, and 7-OH-CBD: 10; THC-COOH, 6-α-OH-CBD and 6-β-OH-CBD: 0.06 LOQ: THC, THC-OH, CBD, 7-COOH-CBD, and 7-OH-CBD: 50; THC-COOH, 6-α-OH-CBD, and 6-β-OH-CBD: 0.2 Injection volume: 1	Lo Faro et al., 2022 [94]
Scalp hair	50	THC, THC-COOH, and CBN	Deuterated water and dichloromethane	1 M NaOH, 90 °C, 15 min	Polymeric strong anion mixed-mode SPE [cyclohexane/ethyl acetate/acetic acid (80:20:5, v/v/v)]	Methanolic HCl and FMP-TS	LC-MS/MS (MRM-ESI+)	Linearity: THC and CBN: 20 to 4000; THC-COOH: 0.2 to 12 LOD: THC and CBN: 2.0; THC-COOH: 0.1 LOQ: THC and CBN: 20.0; THC-COOH: 0.2 Injection volume: 30	Al-Zahrani et al., 2021 [6]
Scalp hair	50	THC, THC-OH, di-THC-OH, THC-COOH, CBN, and CBD	Dichloromethane (thrice)	1 N NaOH, 95 °C, 15 min	SPE [MeOH:formic acid (98:2, v/v)] and SPE [isopropanol:dichloromethane (75:25, v/v)]	N/A	LC-MS/MS (MRM-ESI+)	Linearity: 40 to 20000 LOD: THC, THC-OH, THC-COOH, CBN, and CBD: 40; di-THC-OH: 100 LOQ: THC and CBN: 40; THC-OH, di-THC-OH, THC-COOH, and CBD: 100 Injection volume: 20	Cobo-Golpe et al., 2021 [95]
Scalp and pubic hair	50	THC, THC-COOH, and CBD	Dichloromethane (twice)	10 N NaOH, 75 °C, 1 h	LLE [hexane/ethyl acetate (90:10, v/v)]	N/A	THC and CBD: UHPLC-MS/MS (SRM-ESI+) THC-COOH: UHPLS/MS^3^ (SRM-ESI-)	Linearity: THC and CBD: 20 to 1000; THC-COOH: 0.2 to 10 LOD: THC: 5.3; THC-COOH: 0.07; CBD: 10 LOQ: THC: 10.6; THC-COOH: 0.14; CBD: 20 Injection volume: 5	Gerace et al., 2021 [70]
Scalp hair	50	THC, THC-COOH, CBN, and CBD	Purified water (once) and methanol (twice)	1 M KOH, 70 °C, 1 h	SPE [n-hexane/ethyl acetate/acetic acid (80:18:2, v/v/v).	N/A	THC, CBN and CBD: LC-MS/MS (MRM-ESI+) THC-COOH: LC-MS/MS (MRM-ESI-)	Linearity: THC, CBN and CBD: 25 to 800; THC-COOH: 0.1 to 3.2 LOD: N/A LOQ: THC, CBN and CBD: 25; THC-COOH: 0.1 Injection volume: 10	Schaefer et al., 2021 [96]
Scalp hair	50	THC	Dichloromethane	Acetonitrile, 50 °C, overnight	LLE [(hexane/ethyl acetate (55:45, v/v)] and reversed phase SPE	N/A	N/A	Linearity: N/A LOD: 50 LOQ: 100 Injection volume: N/A	Concheiro et al., 2021 [97]
Beard hair	50	CBD	N/A	1 M NaOH, 95 °C, 10 min	LLE [hexane/ethyl acetate (90:10, v/v)]	BSTFA + 1% TMCS	GC-MS/MS (MRM-EI)	Linearity: 1 to 100 LOD: 1 LOQ: N/A Injection volume: 1	Ameline et al., 2020 [64]
Scalp hair	25	THC	Dichloromethane (twice)	M3^®^ reagent, 100 °C, 1 h	N/A	N/A	UHPLC-MS/MS (MRM-ES+)	Linearity: 25 to 20000 LOD: 2 LOQ: 25 Injection volume: 1	Mannocchi et al., 2020 [98]
Scalp hair	10	THC, THC-COOH, THC-COOH-gluc, CBN, and CBD	Water and acetone (twice)	0.5% formic acid in methanol, 50 ºC, 30 min	LLE (methanol with 0.5% formic acid)	N/A	LC-HRMS (PMR-ESI+)	Linearity: THC, CBN and CBD: 4 to 800; THC-COOH and THC-COOH-gluc: 0.1 to 20 LOD: THC: 1.2; THC-COOH: 0.03; THC-COOH-gluc: 0.02; CBN: 0.7; CBD: 0.8 LOQ: THC, CBN and CBD: 4; THC-COOH and THC-COOH-glu: 0.1 Injection volume: 10	Shin et al., 2020 [99]

Legend: BSTFA [*N*,*O*-Bis(trimethylsilyl)trifluoroacetamide)]; CBD (Cannabidiol); CBN (Cannabinol); di-THC-OH (8-β-11-dihydroxy-THC); EI (Electron ionization); ESI (Electrospray ionization); FMP-TS (2-fluoro-1-methylpyridinium-p-toluenesulfonate); GC-MS/MS (Gas chromatography-tandem mass spectrometry); LC-MS/MS (Liquid chromatography-tandem mass spectrometry); LC-HRMS (Liquid chromatography-high resolution mass spectrometry); LC-MS^3^ (Liquid chromatography-triple stage mass spectrometry); LLE (Liquid-liquid extraction); LOD (Limit of detection); LOQ (Limit of quantification); MRM (Multiple reaction monitoring); N/A (Not available); PRM (Parallel reaction monitoring); SPE (Solid-phase extraction); SRM (Selected reaction monitoring); THC (Δ^9^-tetrahydrocannabinol); THC-COOH (11-nor-9-carboxy-Δ^9^-tetrahydrocannabinol); THC-COOH-gluc (11-nor-9-carboxy-Δ^9^-tetrahydrocannabinol-glucoronide); THC-OH (1-hydroxy-Δ^9^-tetrahydrocannabinol); TMCS (Trimethylchlorosilane); UHPLC-MS/MS (Ultra-high-performance liquid chromatography-tandem mass spectrometry); UHPLC-MS^3^ (Ultra-high-performance liquid chromatography-triple stage mass spectrometry); 6-α-OH-CBD (6-α-hydroxycannabidiol); 6-β-OH-CBD (6-β-hydroxycannabidiol); 7-COOH-CBD (cannabidiol-7-oic acid); 7-OH-CBD (7-hydroxycannabidiol).

### 4.3. Sweat and Exhaled Breath

Sweat analysis has several advantages from an analytical viewpoint. Considering that it is mainly constituted of water (99%), it has fewer impurities than most samples. It is also stable, adulteration is difficult [36,64], and its collection is non-invasive—usually, it is collected by cellulose patches placed on the arm or back of an individual for 7 to 10 days [2,37]. After removal, the saturated patch is treated in an extensive extraction process before being analysed by a chromatographic technique [37,64], which constitutes one of the disadvantages of this sample’s analysis. In addition, there is a possibility of contamination from the collection bulb [36,64].

Several factors can influence the amount of sweat an individual produces every day (which is usually between 300 and 700 mL/day): intense physical activity; emotional, mental, or physical stress; ambient and body temperature; and environmental humidity. These factors, together with the fact that there is usually an uneven distribution of the sweat glands, make it difficult to systematically obtain sweat specimens [64].

There are not many studies in the literature concerning cannabinoid monitorization in sweat samples, especially within the last five years. Analytical methods developed in this time frame for sweat samples are displayed in Table 6.

Ameline et al. [64] were the first team to detect CBD in sweat after the consumption of an oral capsule. CBD was detected in sweat patches for 144 h, and concentrations ranged from 41 to 96 pg/patch. The maximum concentration was achieved 24 h after the administration of the CBD capsule.

Concerning exhaled breath, there is still little information on drugs and their metabolites’ behaviour in this matrix. However, its analysis can be used as a rapid and simple way to detect drug exposure, since its window of detection is 1 to 12 h [2,37]. The fact that the collection of this sample is non-invasive and easy to perform makes it extremely useful to enforcement authorities, especially in DIUD cases [2]. This matrix is mainly constituted by volatile organic compounds and non-volatile compounds present in suspended solid particles [37].

Exhaled breath is collected with collection devices, and, after collection, an organic solvent is usually added. Concerning cannabinoids, THC is the most detected analyte, particularly for early consumption assessment [37]. Analytical methods developed in the last five years for exhaled breath samples are displayed in Table 6.

In 2020, Hubbard et al. [44] developed a rapid and simple LC-MS/MS analytical methodology to quantify THC in breath. A simple methanol elution allowed the team to obtain a LLOQ of 80 pg/pad. This methodology was applied in the previously mentioned study by Hubbard et al. [54] THC was considered an exceptional biomarker for cannabis consumption in breath until 40 min after smoking.

Ameline et al. [64] were the first to detect CBD in this matrix after consumption of an oral capsule. The team was able to detect this analyte until 45 min after administration; reaching concentrations up to 302 pg/filter, a concentration five times lower than that measured for THC.

**Table 6:** Methods for the identification and quantification of cannabinoids in sweat and exhaled breath.

Sample	Collection	Amount (µL)	Analyte(s)	Extraction (Extraction Solvent)	Derivatization	Detection Technique (Acquisition Mode)	Linearity (units) LOD and LOQ (units) Injection Volume (μL)	Reference
Sweat	Collection device (PharmCheck^TM^)	N/A	CBD	LLE [hexane/ethyl acetate (90:10, v/v)]	BSTFA + 1% TMCS	GC-MS/MS (MRM-EI+)	Linearity: 10 to 1000 pg/patch LOD: 10 pg/patch LOQ: N/A Injection volume: 1	Ameline et al., 2020 [64]
Sweat	N/A	N/A	THC and CBD	Methanol elution	N/A	UHPLC-MS/MS (MRM-ESI+)	Linearity: N/A LOD: THC: 0.05 ng/mL; CBD: 0.06 ng/mL LOQ: THC: 0.1 ng/mL; CBD: 0.13 ng/mL Injection volume: 10	Pichini et al., 2020 [47]
Sweat of a fingerprint	Collection device (Drug Screening Cartridge)	N/A	THC	N/A	N/A	UPLC-MS/MS (MRM-ESI+)	Linearity: N/A LOD: N/A LOQ: N/A Injection volume: N/A	Hudson et al., 2019 [100]
Exhaled breath	Collection device (SensAbues AB)	N/A	THC, THCV, THCA, Δ^8^-THC, CBN, CBD, CBDA, CBC, CBG, and CBGA	Methanol elution	N/A	UHPLC-HRMS (HESI+)	Linearity: 2.5 to 100 ng/mL LOD: N/A LOQ: N/A Injection volume: 5	Wurz et al., 2022 [101]
Exhaled breath	Collection device (SensAbues^®^)	N/A	THC	Methanol elution	N/A	LC-MS/MS (MRM-ESI+)	Linearity: ULOQ: 500,000 pg/pad LOD: N/A LOQ: 80 pg/pad Injection volume: 10	Hubbard et al., 2020 [44]
Exhaled breath	Collection device (ExaBreath^®^ DrugTrap)	N/A	CBD	LLE [hexane/ethyl acetate (90:10, v/v)]	BSTFA + 1% TMCS	GC-MS/MS (MRM-EI)	Linearity: 10 to 1000 pg/filter LOD: 10 pg/filter LOQ: N/A Injection volume: 1	Ameline et al., 2020 [64]
Exhaled breath	Collection device	N/A	THC, THC-OH, THC-COOH, CBN, and CBD	Derivatize and shoot	Diazonium solution	LC-MS/MS (MRM-ESI+)	Linearity: 0.1 to 1000 pg/mL LOD: N/A LOQ: THC and CBD: 0.5 pg/mL; THC-OH and THC-COOH: 1 pg/mL; CBN: 0.1 pg/mL Injection volume: 50	Luo et al., 2019 [102]

Legend: BSTFA [*N*,*O*-Bis(trimethylsilyl)trifluoroacetamide)]; CBC (Cannabichromene); CBD (Cannabidiol); CBDA (Cannabidiolic acid); CBG (Cannabigerol); CBGA (Cannabigerolic acid); CBN (Cannabinol); EI (Electron ionization); ESI (Electrospray ionization); GC-MS/MS (Gas chromatography-tandem mass spectrometry); HESI (Heated electrospray ionization); LC-MS/MS (Liquid chromatography-tandem mass spectrometry); LLE (Liquid-liquid extraction); LOD (Limit of detection); LOQ (Limit of quantification); MRM (Multiple reaction monitoring); N/A (Not available); THC (Δ^9^-tetrahydrocannabinol); THCA (Δ^9^-tetrahydrocannabinolic acid); THC-COOH (11-nor-9-carboxy-Δ^9^-tetrahydrocannabinol); THC-OH (1-hydroxy-Δ^9^-tetrahydrocannabinol); THCV (Tetrahydrocannabivarin); TMCS (Trimethylchlorosilane); UHPLC-HRMS (Ultra-high-performance liquid chromatography-high resolution mass spectrometry); UHPLC-MS/MS (Ultra-high-performance liquid chromatography-tandem mass spectrometry); ULOL (Upper limit of linearity); ULOQ (Upper limit of quantification); Δ^8^-THC (Δ^8^-tetrahydrocannabinol).

### 4.4. Other Unconventional Biological Samples

Unconventional biological samples have been thoroughly studied in the last few years. With the help of MS methodologies, small quantities of different xenobiotics can be traced in most matrices; these matrices offer many advantages when compared to the so-called conventional matrices. These specimens are usually studied to provide relevant complementary information, or in cases in which conventional matrices are not available—forensic cases in which body fluids cannot be collected, for example [103].

There are several reasons as to why a team can choose to study these matrices. Their collection procedure is usually non-invasive, many compounds are stable in them, there is usually low possibility for contamination or adulteration, and usually sample preparation is easy and fast. Furthermore, a retrospective analysis can be made when these specimens are studied. Thus, these matrices are usually analysed in forensic, anti-doping, and child custody cases [103].

However, most analytes are usually present at very low concentrations in most unconventional samples, and sample volume is usually low. In addition, there is still a low number of published studies concerning these matrices [103].

Nails, bile fluid, vitreous humor, pericardial fluid, cerumen, meconium, breast milk, placenta, or even blood from the umbilical cord have also been analysed for cannabinoid detection.

Nail analysis can pose as an alternative to hair samples when the latter is not available, since they are too a keratinized matrix. An advantage of nail analysis over hair is that nails are not constituted by melanin and have a continuous and slower growth rate, which allows the detection of lower doses. However, the distribution of cannabinoids in this biological specimen has been scarcely studied. Usually, fingernails have higher concentrations of xenobiotics than toenails and even hair; they are also more prone to external contamination [95].

Bile is a waste fluid and so, like urine, is expected to present higher concentrations of target compounds when compared to matrices such as blood. This matrix is especially important in forensic cases in which blood might not be available [104], since a wide range of metabolites are therein detected [105]. Cannabinoids have recently been added to the list of substances routinely analysed in postmortem samples, such as bile and vitreous humor, but the high complexity of their metabolism and postmortem redistribution makes it difficult to understand their disposition [104].

Vitreous humor is anatomically protected within the eye globe, and as such is more resistant to putrefactive and/or redistribution phenomena, usually common in postmortem analysis. This specimen has been used in toxicology for more than 50 years, mainly in the determination of ethanol. Within the time frame of this review, only two papers involving cannabinoid determination is this specimen were found. Pettersen et al. [106] have analysed several specimens belonging to 39 THC positive cases (in peripheral blood), and only two were positive for the compound in vitreous humor. These same authors have analysed pericardial fluid in some of these cases and did not have any positive findings.

Cerumen, the so-called earwax, is a usually neglected biological matrix when it comes to the study of xenobiotics despite the fact that its analysis may have some advantages. It is not exposed to external contamination, the collection procedures are easy and non-invasive, and the sample is readily available; in addition, substances can be accumulated for longer periods when compared to conventional matrices. However, cerumen does not provide a timeline for consumption, so it should be investigated only as a complementary sample. Concerning cannabinoids, it has been reported that CBN is present alongside THC in cannabis users [107]. Cerumen is a mixture of sebum and sweat, and as such compounds that are excreted in these matrices can be found in the former as well [2].

Meconium is the preferred sample to study prenatal exposure to drugs [2]. This matrix’s collection is non-invasive but can be unavailable for analysis if it is expelled before or during delivery. Additionally, this sample can only be available days or weeks after birth, and there is limited quantity [108]. Meconium has been studied to identify fetal cannabis exposure in the third trimester, since its formation begins between the 12th and the 16th week of gestation [36,109]. THC-OH and THC-COOH are the most abundant compounds detected in this sample [2,110].

Recently, the umbilical cord has been used as an alternative matrix to meconium considering some of the advantages it possesses, such as being an easy and non-invasive collection procedure and yielding large sample volumes. Furthermore, the umbilical cord does not showcase administration of medications after birth, unlike meconium. However, xenobiotics’ disposition in this matrix is not well studied yet [111]. Still, samples can be analysed immediately after birth if there is suspicion of drug abuse [112]. This specimen is formed around the 5th week of gestation [111].

Due to the highly lipophilic characteristic nature of THC, this cannabinoid can cross the placenta and is also able to reach breast milk. Alongside THC, other cannabinoids such as CBN and CBD can accumulate in breast milk since the mammary cell is permeable to lipid-soluble compounds. This matrix is mainly constituted by water, proteins, lipids, and carbohydrates. Thus, cannabinoid extraction from this specimen is challenging considering the solubility of these compounds in its lipidic constituents. This high quantity of lipid contents makes it so that saponification of the samples is usually needed to improve extraction efficiency. There are not many studies concerning cannabinoid disposition in breast milk, which poses a problem not only concerning mothers who use cannabis, but also concerning the safety of human milk banks [113]. This matrix has a relatively wide window of detection since the production of breast milk begins up to weeks before birth [113,114].

The placenta is the least investigated matrix regarding consumption of cannabinoids during pregnancy, and there is not much information regarding this matrix and cannabinoids consumption [97].

Analytical methods developed in the last five years for other unconventional biological samples are displayed in Table 7.

## 5. Conclusions

The development of new analytical methodologies for cannabinoid monitoring in different biological specimens is extremely important considering their high consumption rates in most countries.

Several different analytical techniques have been developed to study these compounds in biological matrices. GC-MS methodologies were, during several years, the most used method for xenobiotics confirmation in biological samples. However, this technique requires expensive and time-consuming sample preparation and derivatization steps, the latter causing deterioration of both the injection liner and the analytical column. This has led to an increase in the development of LC-MS/MS based procedures that generally do not require derivatization steps. This chromatographic technique has a larger field of applications than GC-MS/MS, especially in toxicological laboratories; allows the simultaneous determination of various compounds in the same sample with only one sample preparation and one injection; and, in some cases, samples may be diluted and injected directly into the equipment. These factors increase sensitivity and selectivity and lower LOQs.

The utilization of miniaturized and environmentally friendly systems (considering the AGREE-Analytical GREEnness Metric Approach) will be undoubtedly an enormous asset, since the number of organic solvents is low, being less risky to the analyst as well. Notwithstanding, efforts are still needed concerning the complete automation of those systems.

Regarding biomarkers of cannabis intake, THC and its two main primary and secondary metabolites (THC-OH and THC-COOH, respectively) are usually studied to prove recent consumption. CBD is usually included to allow distinguishing between consumption patterns, since this analyte is only present in medicinal preparations.

Nowadays, it is possible to achieve extremely low LOD in cannabinoid analysis. However, the cut-off value proposed by the SoHT for THC-COOH in hair is still an analytical challenge. Newly developed and more efficient detectors are definitely helping in detecting those low concentrations.

In fact, laboratories are nowadays facing several challenges, such as the determination of cannabinoids in unconventional specimens. Indeed, their concentrations are usually very low even in the usually analysed blood and urine, and therefore one can imagine the even lower concentrations that will appear in samples such as vitreous humour or pericardial fluid. However, the number of studies involving those samples is still too little in order to adequately interpret the results.

The discrimination of Δ^9^-THC isomers, for instance Δ^8^-THC, in the different biological matrices is an additional difficulty for laboratories.

Establishing a biomarker of recent cannabis use to aid in diagnosing DUID situations may be important, for instance to complement behavioural observations. As such, studying cannabinoid profiles in large populations of frequent and occasional users may be an important tool in supporting forensic interpretation, ultimately contributing to the development of scientifically sound laws concerning driving under the influence of cannabis.

The development and validation of analytical methods for cannabinoid screening is an ongoing worldwide project. As cannabis is becoming legalized in a growing number of countries, different analytical methods are developed every year to tackle this reality.

## Figures and Tables

**Table 1:** Methods for the identification and quantification of cannabinoids in whole blood, plasma, and serum.

Sample	Amount (µL)	Analyte(s)	Extraction (Extraction Solvent)	Derivatization	Detection Technique (Acquisition Mode)	Linearity (ng/mL) LOD and LOQ (ng/mL) Injection Volume (μL)	Reference
Whole blood	750	THC, THC-OH, and THC-COOH	QuEChERS (MgSO; NaCl; acetonitrile; D-SPE with C_18_)	HMDS/TMCS/ acetonitrile (1:1:1, v/v/v)	GC-MS/MS (MRM-EI)	Linearity: N/A LOD: THC: 0.008; THC-OH: 0.015; THC-COOH: 0.009 LOQ: N/A Injection volume: 1	Dawidowicz et al., 2022 [40]
Whole blood	250	THC, Δ^8^-THC, THC-OH, and THC-COOH	LLE [hexane/ethyl acetate/methyl-*tert*-butyl-ether (80:10:10, v/v/v)]	N/A	LC-MS/MS (MRM-ESI+)	Linearity: THC: 0.5 to 50; THC-OH: 1.0 to 100; THC-COOH: 5.0 to 500 LOD: THC: 0.13; Δ^8^-THC and THC-OH: 0.25; THC-COOH: 0.31 LOQ: THC and Δ^8^-THC: 0.5; THC-OH: 1; THC-COOH: 5 Injection volume: 10	Chan-Hosokawa et al., 2022 [8]
Whole blood	1000	THC, Δ^8^-THC, THC-COOH, and Δ^8^-THC-COOH	SPE [hexane/ethyl acetate/glacial acetic acid (49:49:2, v/v/v)]	N/A	LC-MS/MS (MRM-ESI+)	Linearity: THC and Δ^8^-THC: 1 to 50; THC-COOH and Δ^8^-THC-COOH: 5 to 250 LOD: THC and Δ^8^-THC: 1; THC-COOH and Δ^8^-THC-COOH: 5 LOQ: THC and Δ^8^-THC: 1; THC-COOH and Δ^8^-THC-COOH: 5.0 Injection volume: 10	Reber et al., 2022 [41]
Whole blood	250	THC, THC-OH, THC-COOH, CBN, and CBD	Automated SPE (acetonitrile)	MSTFA	GC-MS/MS (SRM-EI)	Linearity: THC: 0.3 to 20; THC-OH: 0.3 to 15; THC-COOH: 3 to 150; CBN: 0.2 to 12; CBD: 0.3 to 20 LOD: THC, THC-OH, and CBD: 0.15; THC-COOH: 1; CBN: 0.1 LOQ: THC, THC-OH, and CBD: 0.3; THC-COOH: 3; CBN: 0.2 Injection volume: 1	Frei et al., 2022 [38]
Whole blood	500	THC and THC-COOH	QuEChERS [H_2_O; acetonitrile; MgSO_4_/NaOAc (4:1); primary and secondary amine; MgSO_4_]	N/A	UHPLC-MS/MS (MRM-ESI+)	Linearity: THC: 4 to 400; THC-COOH: 10 to 240 LOD: THC: 1; THC-COOH: 4 LOQ: THC: 4; THC-COOH: 10 Injection volume: 1	Ferrari et al., 2022 [42]
Plasma	200	THC, THC-gluc, THCV, THC-OH, THC-COOH, THC-COOH-gluc, THCV-COOH, CBN, CBD, CBD-gluc, 6-α-OH-CBD, 6-β-OH-CBD, 7-OH-CBD, 7-CBD-COOH, CBDV, CBC, and CBG	One-step protein precipitation [water with 0.2 M ZnSO_4_/methanol (30:70, v/v)]	N/A	HPLC-MS/MS (MRM-APCI+)	Linearity: THC, THCV, THC-COOH, THCV-COOH, CBN, CBD, CBD-gluc, 7-CBD-COOH, CBDV, and CBG: 0.78 to 400; THC-OH, CBC, 6-α-OH-CBD, and 6-β-OH-CBD: 1.56 to 400; 7-OH-CBD: 3.13 to 400; THC-gluc: 0.78 to 200; THC-COOH-gluc: 7.8 to 2000 LOD: N/A LOQ: THC, THC-gluc, THCV, THC-COOH, THCV-COOH, CBN, CBD, CBD-gluc, 7-CBD-COOH, CBDV, and CBG: 0.78; THC-OH, CBC, 6-α-OH-CBD, and 6-β-OH-CBD: 1.56; 7-OH-CBD: 3.13; THC-COOH-gluc: 7.8 Injection volume: 250	Sempio et al., 2021 [43]
Whole blood	200	THC, THCV, THC-OH, THC-COOH, THC-COOH-gluc, CBN, CBD, and CBG	SPE [acetonitrile/isopropanol (90:10, v/v) and acetonitrile/methanol (50:50, v/v) with 2% formic acid]	N/A	THC, THCV, THC-OH, THC-COOH, CBN, CBD and CBG: LC-MS/MS (MRM-ESI+) THC-COOH-gluc: LC-MS/MS (MRM-ESI-)	Linearity: THC, THC-COOH, THC-COOH-gluc, and CBN (ULOQ): 1000; THCV, THC-OH, CBD, and CBG (ULOQ): 100 LOD: N/A LOQ: THC, THCV, CBN, and CBD: 0.5; THC-OH, THC-COOH, and CBG: 1; THC-COOH-gluc: 2 Injection volume: 10	Hubbard et al., 2020 [44]
Whole blood	30	THC and CBD	Online SPE	N/A	UHPLC-MS/MS (SRM-APCI+)	Linearity: 1 to 800 LOD: N/A LOQ: 1 Injection volume: 50	Pigliasco et al., 2020 [45]
Whole blood	200	THC and THC-COOH	Mini-QuEChERS (MgSO_4_; K_2_CO_3_; NaCl; acetonitrile; primary and secondary amine; MgSO_4_)	N/A	UHPLC-MS/MS (MRM-ESI-positive and negative modes by polarity switching)	Linearity: 5 to 6000 LOD: THC: 6.18; THC-COOH: 3.31 LOQ: THC: 18.54; THC-COOH: 3.31 Injection volume: 5	Orfanidis et al., 2020 [46]
Plasma	100	THC, THC-OH, THC-COOH, CBN, and CBD	SALLE [MgSO_4_/NaCl/sodium citrate dihydrate (40:10:10, w/w/w)]	N/A	LC-MS/MS (MRM-ESI)	Linearity: THC, THC-OH, CBN, and CBD: 0.5 to 50; THC-COOH: 1 to 100 LOD: N/A LOQ: THC, THC-OH, CBN, and CBD: 0.5; THC-COOH: 1 Injection volume: 3	da Silva et al., 2020 [39]
Serum	100	THC, THC-gluc, THCA-A, THC-OH, THC-COOH, THC-COOH-gluc, CBD, and CBDA	LLE [acetone:acetonitrile (80:20, v/v)]	N/A	UHPLC-MS/MS (MRM-ESI+)	Linearity: N/A LOD: THC: 0.06; THC-gluc and THC-COOH: 0.09; THCA-A, and CBDA: 0.075; THC-OH: 0.07; THC-COOH-gluc: 0.85; CBD: 0.05 LOQ: THC and THC-OH: 0.12; THC-gluc and THC-COOH: 0.19; THCA-A and CBDA: 0.14; THC-COOH-gluc: 0.17; CBD: 0.13 Injection volume: 10	Pichini et al., 2020 [47]
Whole blood	1000	THC, THC-OH, THC-COOH, and CBD	LLE [hexane/ethyl acetate (90:10, v/v)]	N/A	THC and CBD: LC-HRMS (PRM-ESI+) THC-OH and THC-COOH: LC-HRMS (PRM-ESI-)	Linearity: THC, THC-OH, and CBD: 0.4 to 2; THC-COOH: 2 to 100 LOD: N/A LOQ: THC, THC-OH, and CBD: 0.4; THC-COOH: 2.5 Injection volume: 10	Joye et al., 2020 [48]

Legend: APCI (Atmospheric pressure chemical ionization); CBC (Cannabichromene); CBD (Cannabidiol); CBDA (Cannabidiolic acid); CBD-gluc (Cannabidiol-glucoronide); CBDV (Cannabidivarin); CBG (Cannabigerol); CBN (Cannabinol); EI (Electron ionization); ESI (Electrospray ionization); GC-MS/MS (Gas chromatography-tandem mass spectrometry); HMDS [Bis(trimethylsilyl)amine]; HPLC-MS/MS (High-performance liquid chromatography-tandem mass spectrometry); LC-HRMS (Liquid chromatography-high-resolution mass spectrometry); LC-MS/MS (Liquid chromatography-tandem mass spectrometry); LLE (Liquid-liquid extraction); LOD (Limit of detection); LOQ (Limit of quantification); MRM (Multiple reaction monitoring); MSTFA (*N*-Methyl-*N*-trimethylsilyl-trifluoroacetamide); N/A (Not available); PRM (Parallel reaction monitoring); QuEChERS (Quick, Easy, Cheap, Effective, Rugged and Safe); SALLE (Salting-out assisted liquid-liquid extraction); SPE (Solid-phase extraction); SRM (Selected reaction monitoring); THC (Δ^9^-tetrahydrocannabinol); THCA-A (Δ^9^-tetrahydrocannabinolic acid A); THC-COOH (11-nor-9-carboxy-Δ^9^-tetrahydrocannabinol); THC-COOH-gluc (11-nor-9-carboxy-Δ^9^-tetrahydrocannabinol-glucoronide); THC-gluc (Δ^9^-tetrahydrocannabinol-glucoronide); THC-OH (1-hydroxy-Δ^9^-tetrahydrocannabinol); THCV (Tetrahydrocannabivarin); THCV-COOH (11-nor-9-carboxy-Δ^9^-tetrahydrocannabivarin); TMCS (Trimethylchlorosilane); UHPLC-MS/MS (Ultra-high-performance liquid chromatography-tandem mass spectrometry); ULOQ (Upper limit of quantification); 6-α-OH-CBD (6-α-hydroxycannabidiol); 6-β-OH-CBD (6-β-hydroxycannabidiol); 7-COOH-CBD (Cannabidiol-7-oic acid); 7-OH-CBD (7-hydroxycannabidiol); Δ^8^-THC (Δ^8^-tetrahydrocannabinol); Δ^8^-THC-COOH (11-nor-9-carboxy-Δ^8^-tetrahydrocannabinol).

**Table 2:** Cut-off values for cannabinoids in different biological samples.

Sample	THC (Screening)	THC (Confirmation)	THC-OH	THC-COOH (Screening)	THC-COOH (Confirmation)
Urine	N/A	N/A	N/A	50 ng/mL [59]	15 ng/mL [59,60]
Oral fluid	4 ng/mL [61] 10 ng/mL [62]	2 ng/mL [61,62]	N/A	N/A	N/A
Hair	100 pg/mg [29]	50 pg/mg [29]	0.5 pg/mg [7]	N/A	0.2 pg/mg [29]

Legend: CBD (Cannabidiol); CBN (Cannabinol); N/A (Not available); THC (Δ^9^-tetrahydrocannabinol); THC-COOH (11-nor-9-carboxy-Δ^9^-tetrahydrocannabinol); THC-OH (1-hydroxy-Δ^9^-tetrahydrocannabinol).

**Table 4:** Methods for the identification and quantification of cannabinoids in OF.

Collection	Amount (µL)	Analyte(s)	Extraction (Extraction Solvent)	Derivatization	Detection Technique (Acquisition Mode)	Linearity (ng/mL) LOD and LOQ (ng/mL) Injection Volume (μL)	Reference
Collection device (FLOQSwab^TM^)	500	THC	Online SPE	N/A	UHPLC-MS/MS (MRM-EI+)	Linearity: 1 to 100 LOD: 1 LOQ: 1 Injection volume: 50	Mercier et al., 2022 [85]
Collection device (Quantisal^TM^)	250	THC	LLE [isopropanol/ hexane/ethyl acetate; (50:350, v/v)]	N/A	LC-MS/MS (MRM-ESI+)	Linearity: N/A LOD: 4 LOQ: N/A Injection volume: N/A	Coulter et al., 2022 [86]
N/A	10	THC	Biofluid/methanol (70:30, v/v)	Fast Red RC derivatization reagent	PS-MS/MS (SRM-ESI+)	Linearity: 2 to 250 LOD: 0.78 LOQ: 10 Injection volume: N/A	Borden et al., 2022 [69]
Collection device (Quantisal^TM^)	400	THC, ∆^8^-THC, THCV, THCA-A, THC-OH, THC-COOH, CBN, CBD, CBD-C1, CBDA, CBDV, CB,C and CBG	SPE [acetonitrile/methanol (90:10, v/v)]	N/A	HPLC-MS/MS (MRM-ESI+)	Linearity: THC, ∆^8^-THC, THCV and CBD: 0.10 to 800; THC-OH and THC-COOH: 0.25 to 800; CBN, CBD-C1, CBDV, and CBG: 0.10 to 100; CBDA and CBC: 0.50 to 500; THCA-A: 2.0 to 500 LOD: N/A LOQ: THC, ∆^8^-THC, THCV, CBN, CBD, CBD-C1, CBDV, and CBG: 0.10; THC-OH and THC-COOH: 0.25; CBDA and CBC: 0.50; THCA-A: 2.00 Injection volume: 6	Lin et al., 2021 [79]
Collection device (Quantisal^TM^)	1000	THC, ∆^8^-THC, CBN and CBD	SPE [ethyl acetate and hexane/ethyl acetate/acetic acid (88:10:2, v/v/v)]	N/A	LC-MS/MS (MRM-ESI+)	Linearity: 1 to 100 LOD: THC: 0.13; ∆^8^-THC: 0.68; CBN: 1.09; CBD: 0.47 LOQ: N/A Injection volume: 20	Coulter et al., 2021 [83]
Passive drool	250	THC and CBD	LLE [methanol/ acetonitrile (80:20, v/v)]	N/A	UHPLC-MS/MS (SMR-ESI+)	Linearity: 1 to 15 LOD: 0.5 LOQ: 1 Injection volume: 2	Gerace et al., 2021 [70]
DOFS	50	THC and CBD	Methanol/acetonitrile (50:50, v/v)	N/A	LC-MS/MS (dMRM-ESI+)	Linearity: N/A LOD: THC: 2; CBD: 4 LOQ: N/A Injection volume: 1	Gorziza et al., 2021 [84]
Collection device (NeoSal^TM^)	1000	CBD	LLE [hexane/ethyl acetate (90:10, v/v)]	BSTFA + 1% TMCS	GC-MS/MS (MRM-EI)	Linearity: 0.01 to 100 LOD: 0.01 LOQ: N/A Injection volume: 1	Ameline et al., 2020 [64]
Collection device (Quantisal^TM^)	500	THC	LLE (saturated Na_2_B_4_O_7_ aqueous solution and MTBE)	N/A	LC-MS/MS (MRM-ESI+)	Linearity: N/A LOD: 1 LOQ: N/A Injection volume: 2	da Cunha et al., 2020 [87]
N/A	100	THC, THC-gluc, THCA-A, THC-OH, THC-COOH, THC-COOH-gluc, CBD, and CBDA	LLE [acetone:acetonitrile (80:20, v/v)]	N/A	UHPLC-MS/MS (MRM-ESI+)	Linearity: N/A LOD: THC: 0.05; THC-gluc and THC-COOH-gluc: 0.075; THCA-A and CBDA: 0.07; THC-OH: 0.065; THC-COOH: 0.08; CBD: 0.04 LOQ: THC and CBD: 0.12; THC-gluc: 0.15; THCA-A and THC-OH: 0.13; THC-COOH: 0.19; THC-COOH-gluc: 0.16; CBDA: 0.14 Injection volume: 10	Pichini et al., 2020 [47]

Legend: BSTFA [*N*,*O*-Bis(trimethylsilyl)trifluoroacetamide)]; CBC (Cannabichromene); CBD (cannabidiol); CBDA (Cannabidiolic acid); CBD-C1 (Cannabidiorcol); CBDV (Cannabidivarin); CBG (Cannabigerol); CBN (Cannabinol); dMRM (Dynamic multiple reaction monitoring); DOFS (Dried oral fluid spots); EI (Electron ionization); ESI (Electrospray ionization); GC-MS/MS (Gas chromatography-tandem mass spectrometry); HPLC-MS/MS (High-performance liquid chromatography-tandem mass spectrometry); LC-MS/MS (Liquid chromatography-tandem mass spectrometry); LLE (Liquid-liquid extraction); LOD (Limit of detection); LOQ (Limit of quantification); MRM (Multiple reaction monitoring); MTBE (Methyl tertiary butyl ether); N/A (Not available); PS-MS/MS (Paper spray-tandem mass spectrometry); SPE (Solid-phase extraction); SRM (Selected reaction monitoring); THC (Δ^9^-tetrahydrocannabinol); THCA-A (Δ^9^-tetrahydrocannabinolic acid A); THC-COOH (11-nor-9-carboxy-Δ^9^-tetrahydrocannabinol); THC-COOH-gluc (11-nor-9-carboxy-Δ^9^-tetrahydrocannabinol-glucoronide); THC-gluc (Δ^9^-tetrahydrocannabinol-glucoronide); THC-OH (1-hydroxy-Δ^9^-tetrahydrocannabinol); THCV (tetrahydrocannabivarin); TMCS (Trimethylchlorosilane); UHPLC-MS/MS (Ultra-high-performance liquid chromatography-tandem mass spectrometry); Δ^8^-THC (Δ^8^-tetrahydrocannabinol).

**Table 7:** Methods for the identification and quantification of cannabinoids in other unconventional biological samples.

Sample	Amount (Units)	Analyte(s)	Washing	Digestion	Extraction (Extraction Solvent)	Derivatization	Detection Technique (Acquisition Mode)	Linearity (Units) LOD and LOQ (Units) Injection Volume (μL)	Reference
Nails	50 mg	THC	Water and acetone (twice)	N/A	Acetonitrile: mobile phase A (1:1, v/v)	N/A	UHPLC-MS/MS (MRM-ESI+)	Linearity: 5 to 2000 pg/mg LOD: 0.9946 pg/mg LOQ: 25 pg/mg Injection volume: 1	Liu et al., 2022 [115]
Nails	30 mg	THC, THC-OH, di-THC-OH, THC-COOH, CBN and CBD	Dichloromethane (five washes)	1 N NaOH, 95 °C, 15 min	SPE [MeOH:formic acid (98:2, v/v)]	N/A	LC-MS/MS (MRM-ESI+)	Linearity: 10 to 20,000 pg/mg LOD: THC: 10 pg/mg; THC-OH, di-THC-OH, and CBD: 100 pg/mg; THC-COOH: 50 pg/mg; CBN: 20 pg/mg LOQ: THC and CBD: 20 pg/mg; THC-OH, di-THC-OH, THC-COOH and CBN: 100 pg/mg Injection volume: 20	Cobo-Golpe et al., 2021 [95]
Nails	25 mg	THC	Dichloromethane (twice)	M3^®^ reagent, 100 °C, 1 h	N/A	N/A	UHPLC-MS/MS (MRM-ES+)	Linearity: 20 to 1000 pg/mg LOD: 2 pg/mg LOQ: 20 pg/mg Injection volume: 1	Mannocchi et al., 2020 [98]
Nails	25 mg	THC	Dichloromethane	VMA-TM3 reagent, 100 °C, 1 h	SPE (multimatrix eluent)	N/A	UHPLC-MS/MS (MRM-ESI-)	Linearity: 100 to 50,000 pg/mg LOD: 30 pg/mg LOQ: 100 pg/mg Injection volume: 1	Busardò et al., 2020 [116]
Bile	1 mL	THC, THC-OH, and THC-COOH	N/A	N/A	SPE [n-hexane/ethyl acetate (50:50, v/v)]	N/A	LC-MS/MS (MRM)	Linearity: 0.5 to 1000 ng/mL LOD: THC and THC-OH: 1.2 ng/mL; THC-COOH: 1.1 ng/mL LOQ: 2 ng/mL Injection volume: 1	Al-Asmari et al., 2019 [104]
Pericardial fluid	N/A	THC and CBD	N/A	N/A	LLE (hexane)	BSTFA in acetonitrile (1:2)	GC-MS (SIM, EI)	Linearity: N/A LOD: 0.02 ng/mL LOQ: N/A Injection volume: 2	Pettersen et al., 2021 [106]
Vitreous humor	0.5 mL	THC and CBD	N/A	N/A	LLE (hexane)	BSTFA in acetonitrile (1:2)	GC-MS (SIM, EI)	Linearity: N/A LOD: 0.02 ng/mL LOQ: N/A Injection volume: 2	Pettersen et al., 2021 [106]
Vitreous humor	1 mL	THC, THC-OH, and THC-COOH	N/A	N/A	SPE [n-hexane/ethyl acetate (50:50, v/v)]	N/A	LC-MS/MS (MRM)	Linearity: 0.5 to 1000 ng/mL LOD: THC: 0.7 ng/mL; THC-OH: 0.6 ng/mL; THC-COOH: 0.8 ng/mL LOQ: 1 ng/mL Injection volume: 1	Al-Asmari et al., 2019 [104]
Cerumen	N/A	THC, THC-OH, THC-COOH, CBN, and CBD	N/A	N/A	Acetonitrile with 1% acetic acid	N/A	UHPLC-MS/MS (MRM-ESI+)	Linearity: 100 to 15,000 pg/mg LOD: THC: 0.038 pg/mg; THC-OH: 0.075 pg/mg; THC-COOH: 0.057 pg/mg; CBN: 0.046 pg/mg; CBD: 0.013 pg/mg LOQ: THC: 0.113 pg/mg; THC-OH: 0.225 pg/mg; THC-COOH: 0.170 pg/mg; CBN: 0.139 pg/mg; CBD: 0.040 pg/mg Injection volume: 10	Nicolaou et al., 2021 [107]
Meconium	300 mg	THC, THC-OH, and THC-COOH	N/A	N/A	SPE [acetonitrile/methanol (90/10, v/v).]	N/A	LC-HRMS (MRM-HESI+)	Linearity: 5 to 100 pg/mg LOD: 5 pg/mg LOQ: N/A Injection volume: 5	Hernandez et al., 2022 [117]
Meconium	250 mg	THC, THC-gluc, THC-OH, di-THC-OH, THC-COOH, THC-COOH-gluc, CBN, and CBD	N/A	N/A	Mixed mode cation-exchange SPE	N/A	N/A	Linearity: N/A LOD: 1 to 2 ng/g LOQ: THC, THC-OH, di-THC-OH, THC-COOH, THC-COOH-gluc, CBN, and CBD: 4 ng/g; THC-gluc: 10 ng/g Injection volume: N/A	Concheiro et al., 2021 [97]
Meconium	250 mg	THC, THCA, THC-OH, CBN and CBD	N/A	N/A	SPE [hexane/ethyl acetate (90:10, v/v) with 2% acetic acid]	N/A	LC-MS/MS (MRM-ESI-)	Linearity: 5 to 1000 ng/g LOD: N/A LOQ: 5 ng/g Injection volume: 5	Jensen et al., 2019 [111]
Meconium	500 mg	THC-COOH	N/A	N/A	ASE (0.4 mol/L sodium hydroxide) and SPE [hexane/ethyl acetate/acetone/glacial acetic acid (54:18:27:1, v/v/v/v)]	MTBSTFA	GC-MS (SIM-EI)	Linearity: 10 to 500 ng/g LOD: 5 ng/g LOQ: 10 ng/g Injection volume: 2	Mantovani et al., 2018 [118]
Umbilical cord	1 g	THC, THCA, THC-OH, and CBN	N/A	N(A	SPE	N/A	LC-MS/MS (MRM-ESI-)	Linearity: 0.2 to 10.0 ng/g LOD: N/A LOQ: 0.2 Injection volume: 5	Jensen et al., 2019 [111]
Umbilical cord	1 g	THC, THC-OH, THC-COOH, and CBN	N/A	N/A	SPE (2% acetic acid SPE elution buffer)	N/A	LC-MS/MS (MRM-ESI-)	Linearity: 0.2 to 5 ng/g LOD: N/A LOQ: N/A Injection volume: 40	Wu et al., 2019 [112]
Umbilical cord	N/A	THC-COOH	N/A	N/A	SPE	N/A	LC-MS/MS	Linearity: N/A LOD: 0.10 ng/g LOQ: N/A Injection volume: N/A	Metz et al., 2021 [119]
Umbilical cord	0.5 g	THC, THC-gluc, THC-OH, di-THC-OH, THC-COOH, THC-COOH-gluc, and CBD	N/A	N/A	SPE [dichloromethane/isopropanol (30:70, v/v)]	N/A	LC-MS/MS (MRM)	Linearity: THC, THC-COOH, and CBD: 7 to 200 ng/g; THC-OH and di-THC-OH: 10 to 200 ng/g; THC-gluc: 1 to 20 ng/g; THC-COOH-gluc: 1 to 200 ng/g LOD: THC, THC-COOH, and CBD: 7 ng/g; THC-OH and di-THC-OH: 10 ng/g; THC-gluc and THC-COOH-gluc: 1 ng/g LOQ: THC, THC-COOH, and CBD: 7 ng/g; THC-OH and di-THC-OH: 10 ng/g; THC-gluc and THC-COOH-gluc: 1 ng/g Injection volume: N/A	Kim et al., 2018 [120]
Umbilical cord	1 g	THC, THC-OH, THC-COOH, and CBN	N/A	N/A	SPE (2% acetic acid in methanol)	N/A	LC-MS/MS (MRM-ESI-)	Linearity: 0.2 to 30 ng/g LOD: 0.1 ng/g LOQ: 0.2 ng/g Injection volume: 50	Wu et al., 2018 [121]
Umbilical cord	0.5 g	THC, THC-gluc, THC-OH, di-THC-OH, THC-COOH, THC-COOH-gluc, and CBD	N/A	N/A	Mixed mode cation-exchange SPE	N/A	N/A	Linearity: N/A LOD: THC, THC-COOH, and CBD: 7 ng/g; THC-gluc and THC-COOH-gluc: 1 ng/g; THC-OH and di-THC-OH: 10 ng/g LOQ: THC, THC-COOH, and CBD: 7 ng/g; THC-gluc and THC-COOH-gluc: 1 ng/g; THC-OH and di-THC-OH: 10 ng/g Injection volume: N/A	Concheiro et al., 2021 [97]
Breast milk	200 µL	THC, THC-gluc, THCV, THC-OH, THC-COOH, THC-COOH-gluc, THCV-COOH, CBN, CBD, CBDV, CBC, and CBG	N/A	N/A	One-step protein precipitation [water with 0.2 M ZnSO_4_/methanol (30:70, v/v)]	N/A	HPLC-MS/MS (MRM+)	Linearity: THC, THCV, THC-OH, THC-COOH, THCV-COOH, CBN, CBD, CBDV, CBC, and CBG: 0.39 to 400 ng/mL; THC-gluc: 0.04 to 40 ng/mL; THC-COOH-gluc: 1.95 to 2000 ng/mL LOD: N/A LOQ: THC, THCV, CBD, CBDV and CBG: 0.78 ng/mL; THC-gluc: 1.25 ng/mL; THC-OH and THCV-COOH: 1.56 ng/mL; THC-COOH: 0.39 ng/mL; THC-COOH-gluc: 7.8 ng/mL; CBN and CBC: 3.13 ng/mL Injection volume: 50	Sempio et al., 2021 [113]
Breast milk	750 µL	THC, CBN, and CBD	N/A	N/A	QuEChERS (1 N hydrochloric acid; roQTM extraction salt; acetonitrile; D-SPE)	N/A	UPLC-MS/MS (MRM-ESI+)	Linearity: 1 to 100 ng/mL LOD: N/A LOQ: 0.9 ng/mL Injection volume: 5	Ramnarine et al., 2019 [114]
Breast milk	N/A	THC, THC-OH, CBN, and CBD	N/A	N/A	N/A	N/A	THC, THC-OH and and CBD: LC-MS (ESI+) CBN: LC-MS (ESI-)	Linearity: N/A LOD: N/A LOQ: N/A Injection volume: N/A	Bertrand et al., 2018 [122]
Placenta	0.5 g	THC, THC-gluc, THC-OH, di-THC-OH, THC-COOH, THC-COOH-gluc, CBN, and CBD	N/A	N/A	Mixed mode cation-exchange SPE [(dichloromethane/isopropanol (50:50 v/v)]	N/A	N/A	Linearity: THC, THC-COOH, CBN and CBD: 5 to 100 ng/g; THC-gluc: 0.5 to 20 ng/g; THC-OH and di-THC-OH: 20 to 100 ng/g; THC-COOH-gluc: 0.5 to 100 ng/g LOD: THC, THC-COOH, CBN, and CBD: 5 ng/g; THC-gluc and THC-COOH-gluc: 0.5 ng/g; THC-OH and di-THC-OH: 20 ng/g LOQ: THC, THC-COOH, CBN, and CBD: 5 ng/g; THC-gluc and THC-COOH-gluc: 0.5 ng/g; THC-OH and di-THC-OH: 20 ng/g Injection volume: N/A	Concheiro et al., 2021 [97]

Legend: ASE (Accelerated solvent extraction); CBC (Cannabichromene); CBD (Cannabidiol); CBDV (Cannabidivarin); CBG (Cannabigerol); CBN (Cannabinol); di-THC-OH (8-β-11-dihydroxy-THC); EI (Electron ionization); ESI (Electrospray ionization); GC-MS (Gas chromatography-mass spectrometry); HESI (Heated electrospray ionization); HPLC-MS/MS (High-performance liquid chromatography-tandem mass spectrometry); LC-HRMS (Liquid chromatography-high resolution mass spectrometry); LC-MS/MS (Liquid chromatography-tandem mass spectrometry); LOD (Limit of detection); LOQ (Limit of quantification); MRM (Multiple reaction monitoring); MTBSTFA (*N*-Methyl-*N*-tert-butyldimethylsilyltrifluoroacetamide); N/A (Not available); QuEChERS (Quick, Easy, Cheap, Effective, Rugged and Safe); SIM (Selected ion monitoring); SPE (Solid-phase extraction); THC (Δ^9^-tetrahydrocannabinol); THCA (Δ^9^-tetrahydrocannabinolic acid); THC-COOH (11-nor-9-carboxy-Δ^9^-tetrahydrocannabinol); THC-COOH-gluc (11-nor-9-carboxy-Δ^9^-tetrahydrocannabinol-glucoronide); THC-gluc (Δ^9^-tetrahydrocannabinol-glucoronide); THCV-COOH (11-nor-9-carboxy-Δ^9^-tetrahydrocannabivarin); THC-OH (1-hydroxy-Δ^9^-tetrahydrocannabinol); THCV (Tetrahydrocannabivarin); UHPLC-MS/MS (Ultra-high-performance liquid chromatography-tandem mass spectrometry); UPLC-MS/MS (Ultra-performance liquid chromatography-tandem mass spectrometry).

## Data Availability

Not applicable.

## References

[1] Gonçalves J., Rosado T., Soares S., Simão A., Caramelo D., Luís Â., Fernández N., Barroso M., Gallardo E., Duarte A. (2019). Cannabis and Its Secondary Metabolites: Their Use as Therapeutic Drugs, Toxicological Aspects, and Analytical Determination. Medicines.

[2] Nicolaou A.G., Christodoulou M.C., Stavrou I.J., Kapnissi-Christodoulou C.P. (2021). Analysis of Cannabinoids in Conventional and Alternative Biological Matrices by Liquid Chromatography: Applications and Challenges. J. Chromatogr. A.

[3] Cho H.S., Cho B., Sim J., Baeck S.K., In S., Kim E. (2019). Detection of 11-nor-9-Carboxy-Tetrahydrocannabinol in the Hair of Drug Abusers by LC–MS/MS Analysis. Forensic Sci. Int..

[4] Lucas C.J., Galettis P., Schneider J. (2018). The Pharmacokinetics and the Pharmacodynamics of Cannabinoids. Br. J. Clin. Pharmacol..

[5] Grotenhermen F. (2003). Pharmacokinetics and Pharmacodynamics of Cannabinoids. Clin. Pharmacokinet..

[6] Al-Zahrani M.A., Al-Asmari A.I., Al-Zahrani F.F., Torrance H.J., Watson D.G. (2021). Quantification of Cannabinoids in Human Hair Using a Modified Derivatization Procedure and Liquid Chromatography–Tandem Mass Spectrometry. Drug Test. Anal..

[7] Casati S., Angeli I., Ravelli A., Del Fabbro M., Minoli M., Orioli M. (2019). 11-OH-THC in Hair as Marker of Active Cannabis Consumption: Estimating a Reliable Cut-off by Evaluation of 672 THC-Positive Hair Samples. Forensic Sci. Int..

[8] Chan-Hosokawa A., Nguyen L., Lattanzio N., Adams W.R. (2022). Emergence of Delta-8 Tetrahydrocannabinol in DUID Investigation Casework: Method Development, Validation and Application. J. Anal. Toxicol..

[9] Ashton C.H. (1999). Adverse Effects of Cannabis and Cannabinoids. Br. J. Anaesth..

[10] Hall W. (1994). The Health and Psychological Effects of Cannabis Use. Curr. Issues Crim. Justice.

[11] Desrosiers N.A., Huestis M.A. (2019). Oral Fluid Drug Testing: Analytical Approaches, Issues and Interpretation of Results. J. Anal. Toxicol..

[12] European Monitoring Centre for Drugs and Drug Addiction (2022). European Drug Report 2022: Trends and Developments.

[13] Guo T.-T., Zhang J.-C., Zhang H., Liu Q.-C., Zhao Y., Hou Y.-F., Bai L., Zhang L., Liu X.-Q., Liu X.-Y. (2017). Bioactive Spirans and Other Constituents from the Leaves of Cannabis Sativa f. Sativa. J. Asian Nat. Prod. Res..

[14] Huestis M.A., Pertwee R.G. (2005). Pharmacokinetics and Metabolism of the Plant Cannabinoids, ∆9-Tetrahydrocannibinol, Cannabidiol and Cannabinol. Cannabinoids.

[15] United Nations (1962). Single Convention on Narcotic Drugs, 1961—As Amended by the 1972 Protocol Amending the Single Convention on Narcotic Drugs, 1961.

[16] Kataoka H., Saito K. (2011). Recent Advances in SPME Techniques in Biomedical Analysis. J. Pharm. Biomed. Anal..

[17] Namdar D., Mazuz M., Ion A., Koltai H. (2018). Variation in the Compositions of Cannabinoid and Terpenoids in Cannabis Sativa Derived from Inflorescence Position along the Stem and Extraction Methods. Ind. Crops Prod..

[18] Richins R.D., Rodriguez-Uribe L., Lowe K., Ferral R., O’Connell M.A. (2018). Accumulation of Bioactive Metabolites in Cultivated Medical Cannabis. PLoS ONE.

[19] Gul W., Gul S.W., Radwan M.M., Wanas A.S., Mehmedic Z., Khan I.I., Sharaf M.H.M., ElSohly M.A. (2015). Determination of 11 Cannabinoids in Biomass and Extracts of Different Varieties of Cannabis Using High-Performance Liquid Chromatography. J. AOAC Int..

[20] Spindle T.R., Cone E.J., Schlienz N.J., Mitchell J.M., Bigelow G.E., Flegel R., Hayes E., Vandrey R. (2019). Acute Pharmacokinetic Profile of Smoked and Vaporized Cannabis in Human Blood and Oral Fluid. J. Anal. Toxicol..

[21] Sharma P., Murthy P., Bharath M.M.S. (2012). Chemistry, Metabolism, and Toxicology of Cannabis: Clinical Implications. Iran. J. Psychiatry.

[22] Mano-Sousa B.J., Maia G.A.S., Lima P.L., Campos V.A., Negri G., Chequer F.M.D., Duarte-Almeida J.M. (2021). Color Determination Method and Evaluation of Methods for the Detection of Cannabinoids by Thin-Layer Chromatography (TLC). J. Forensic Sci..

[23] Grijó D.R., Olivo J.E., da Motta Lima O.C. (2021). Simple Chemical Tests to Identify Cannabis Derivatives: Redefinition of Parameters and Analysis of Concepts. J. Forensic Sci..

[24] Huang S., Qiu R., Fang Z., Min K., Van Beek T.A., Ma M., Chen B., Zuilhof H., Salentijn G.I.J. (2022). Semiquantitative Screening of THC Analogues by Silica Gel TLC with an Ag(I) Retention Zone and Chromogenic Smartphone Detection. Anal. Chem..

[25] Wang Y.-H., Avula B., Elsohly M.A., Radwan M.M. (2017). Quantitative Determination of Δ9-THC, CBG, CBD, Their Acid Precursors and Five Other Neutral Cannabinoids by UHPLC-UV-MS. Planta Med..

[26] De Backer B., Debrus B., Lebrun P., Theunis L., Dubois N., Decock L., Verstraete A., Hubert P., Charlier C. (2009). Innovative Development and Validation of an HPLC/DAD Method for the Qualitative and Quantitative Determination of Major Cannabinoids in Cannabis Plant Material. J. Chromatogr. B Anal. Technol. Biomed. Life Sci..

[27] Cardenia V., Gallina Toschi T., Scappini S., Rubino R.C., Rodriguez-Estrada M.T. (2018). Development and Validation of a Fast Gas Chromatography/Mass Spectrometry Method for the Determination of Cannabinoids in Cannabis Sativa L.. J. Food Drug Anal..

[28] Mercolini L., Mandrioli R., Protti M., Conti M., Serpelloni G., Raggi M.A. (2013). Monitoring of Chronic Cannabis Abuse: An LC-MS/MS Method for Hair Analysis. J. Pharm. Biomed. Anal..

[29] Cooper G.A.A., Kronstrand R., Kintz P. (2012). Society of Hair Testing Guidelines for Drug Testing in Hair. Forensic Sci. Int..

[30] Gorziza R.P., Duarte J.A., González M., Arroyo-Mora L.E., Limberger R.P. (2021). A Systematic Review of Quantitative Analysis of Cannabinoids in Oral Fluid. J. Forensic Sci..

[31] Ramzy V., Priefer R. (2021). THC Detection in the Breath. Talanta.

[32] Mirzaei H., O’Brien A., Tasnim N., Ravishankara A., Tahmooressi H., Hoorfar M. (2020). Topical Review on Monitoring Tetrahydrocannabinol in Breath. J. Breath Res..

[33] Ahmad S.M., Gonçalves O.C., Oliveira M.N., Neng N.R., Nogueira J.M.F. (2021). Application of Microextraction-Based Techniques for Screening-Controlled Drugs in Forensic Context—a Review. Molecules.

[34] De Giovanni N., Marchetti D. (2020). A Systematic Review of Solid-Phase Microextraction Applications in the Forensic Context. J. Anal. Toxicol..

[35] Karschner E.L., Swortwood-Gates M.J., Huestis M.A. (2020). Identifying and Quantifying Cannabinoids in Biological Matrices in the Medical and Legal Cannabis Era. Clin. Chem..

[36] Gallardo E., Queiroz J.A. (2008). The Role of Alternative Specimens in Toxicological Analysis. Biomed. Chromatogr..

[37] Puiu M., Bala C. (2022). Affinity Assays for Cannabinoids Detection: Are They Amenable to On-Site Screening?. Biosensors.

[38] Frei P., Frauchiger S., Scheurer E., Mercer-Chalmers-Bender K. (2022). Quantitative Determination of Five Cannabinoids in Blood and Urine by Gas Chromatography Tandem Mass Spectrometry Applying Automated On-Line Solid Phase Extraction. Drug Test. Anal..

[39] da Silva C.P., Dalpiaz L.P.P., Gerbase F.E., Muller V.V., Cezimbra da Silva A., Lizot L.F., Hahn R.Z., da Costa J.L., Antunes M.V., Linden R. (2020). Determination of Cannabinoids in Plasma Using Salting-out-Assisted Liquid–Liquid Extraction Followed by LC–MS/MS Analysis. Biomed. Chromatogr..

[40] Dawidowicz A.L., Dybowski M.P., Rombel M., Typek R. (2022). Oleamide as Analyte Protectant in GC Analysis of THC and Its Metabolites in Blood. J. Pharm. Biomed. Anal..

[41] Reber J.D., Karschner E.L., Seither J.Z., Knittel J.L., Dozier K.V., Walterscheid J.P. (2022). An Enhanced LC-MS-MS Technique for Distinguishing Δ8- and Δ9-Tetrahydrocannabinol Isomers in Blood and Urine Specimens. J. Anal. Toxicol..

[42] Ferrari Júnior E., Caldas E.D. (2022). Determination of New Psychoactive Substances and Other Drugs in Postmortem Blood and Urine by UHPLC–MS/MS: Method Validation and Analysis of Forensic Samples. Forensic Toxicol..

[43] Sempio C., Almaraz-Quinones N., Jackson M., Zhao W., Wang G.S., Liu Y., Leehey M., Knupp K., Klawitter J., Christians U. (2022). Simultaneous Quantification of 17 Cannabinoids by LC-MS-MS in Human Plasma. J. Anal. Toxicol..

[44] Hubbard J.A., Smith B.E., Sobolesky P.M., Kim S., Hoffman M.A., Stone J., Huestis M.A., Grelotti D.J., Grant I., Marcotte T.D. (2020). Validation of a Liquid Chromatography Tandem Mass Spectrometry (LC-MS/MS) Method to Detect Cannabinoids in Whole Blood and Breath. Clin. Chem. Lab. Med..

[45] Pigliasco F., Barco S., Dubois S., Marchese F., Striano P., Lomonaco T., Mattioli F., Tripodi G., Cangemi G. (2020). Cannabidiol Determination on Peripheral Capillary Blood Using a Microsampling Method and Ultra-High-Performance Liquid Chromatography Tandem Mass Spectrometry with on-Line Sample Preparation. Molecules.

[46] Orfanidis A., Gika H.G., Theodoridis G., Mastrogianni O., Raikos N. (2021). A UHPLC-MS-MS Method for the Determination of 84 Drugs of Abuse and Pharmaceuticals in Blood. J. Anal. Toxicol..

[47] Pichini S., Mannocchi G., Gottardi M., Pérez-Acevedo A.P., Poyatos L., Papaseit E., Pérez-Mañá C., Farré M., Pacifici R., Busardò F.P. (2020). Fast and Sensitive UHPLC-MS/MS Analysis of Cannabinoids and Their Acid Precursors in Pharmaceutical Preparations of Medical Cannabis and Their Metabolites in Conventional and Non-Conventional Biological Matrices of Treated Individual. Talanta.

[48] Joye T., Widmer C., Favrat B., Augsburger M., Thomas A. (2021). Parallel Reaction Monitoring-Based Quantification of Cannabinoids in Whole Blood. J. Anal. Toxicol..

[49] Rahman M.M., Abd El-Aty A.M., Shim J.H. (2013). Matrix Enhancement Effect: A Blessing or a Curse for Gas Chromatography?-A Review. Anal. Chim. Acta.

[50] Fujiyoshi T., Ikami T., Sato T., Kikukawa K., Kobayashi M., Ito H., Yamamoto A. (2016). Evaluation of the Matrix Effect on Gas Chromatography - Mass Spectrometry with Carrier Gas Containing Ethylene Glycol as an Analyte Protectant. J. Chromatogr. A.

[51] Sugitate K., Anazawa H., Nakamura S., Orikata N., Mizukoshi K., Nakamura M., Toriba A., Hayakawa K. (2012). Decrease in the Matrix Effect of GC/MS by a Gold-Plated Ion Source. J. Pestic. Sci..

[52] Rodríguez-Ramos R., Lehotay S.J., Michlig N., Socas-Rodríguez B., Rodríguez-Delgado M.Á. (2020). Critical Review and Re-Assessment of Analyte Protectants in Gas Chromatography. J. Chromatogr. A.

[53] Sørensen L.K., Hasselstrøm J.B. (2017). Sensitive Determination of Cannabinoids in Whole Blood by LC–MS-MS After Rapid Removal of Phospholipids by Filtration. J. Anal. Toxicol..

[54] Hubbard J.A., Hoffman M.A., Ellis S.E., Sobolesky P.M., Smith B.E., Suhandynata R.T., Sones E.G., Sanford S.K., Umlauf A., Huestis M.A. (2021). Biomarkers of Recent Cannabis Use in Blood, Oral Fluid and Breath. J. Anal. Toxicol..

[55] Meier S.I., Koelzer S.C., Schubert-Zsilavecz M., Toennes S.W. (2017). Analysis of Drugs of Abuse in Cerumen - Correlation of Postmortem Analysis Results with Those for Blood, Urine and Hair. Drug Test. Anal..

[56] Gallardo E., Barroso M., Queiroz J.A. (2009). LC-MS: A Powerful Tool in Workplace Drug Testing. Drug Test. Anal..

[57] Samyn N., Van Haeren C. (2000). On-Site Testing of Saliva and Sweat with Drugwipe and Determination of Concentrations of Drugs of Abuse in Saliva, Plasma and Urine of Suspected Users. Int. J. Legal Med..

[58] Rosendo L.M., Rosado T., Oliveira P., Simão A.Y., Margalho C., Costa S., Passarinha L.A., Barroso M., Gallardo E. (2022). The Determination of Cannabinoids in Urine Samples Using Microextraction by Packed Sorbent and Gas Chromatography-Mass Spectrometry. Molecules.

[59] Substance Abuse and Mental Health Services Administration, Department of Health and Human Services (2022). Mandatory Guidelines for Federal Workplace Drug Testing Programs—Urine. Fed. Regist. Dly. J. United States Gov..

[60] Taskinen S., Beck O., Bosch T., Brcak M., Carmichael D., Fucci N., George C., Piper M., Salomone A., Schielen W. (2017). European Guidelines for Workplace Drug Testing in Urine. Drug Test. Anal..

[61] Substance Abuse and Mental Health Services Administration, Department of Health and Human Services (2019). Mandatory Guidelines for Federal Workplace Drug Testing Programs—Oral/Fluid. Fed. Regist. Dly. J. United States Gov..

[62] Brcak M., Beck O., Bosch T., Carmichael D., Fucci N., George C., Piper M., Salomone A., Schielen W., Steinmeyer S. (2018). European Guidelines for Workplace Drug Testing in Oral Fluid. Drug Test. Anal..

[63] Morisue Sartore D., Costa J.L., Lanças F.M., Santos-Neto Á.J. (2022). Packed In-Tube SPME–LC–MS/MS for Fast and Straightforward Analysis of Cannabinoids and Metabolites in Human Urine. Electrophoresis.

[64] Ameline A., Raul J.S., Kintz P. (2020). Characterization of Cannabidiol in Alternative Biological Specimens and Urine, after Consumption of an Oral Capsule. J. Anal. Toxicol..

[65] Bergamaschi M.M., Barnes A., Queiroz R.H.C., Hurd Y.L., Huestis M.A. (2013). Impact of Enzymatic and Alkaline Hydrolysis on CBD Concentration in Urine. Anal. Bioanal. Chem..

[66] Kraemer M., Broecker S., Madea B., Hess C. (2019). Decarbonylation: A Metabolic Pathway of Cannabidiol in Humans. Drug Test. Anal..

[67] Rodrigues L.C., Kahl J.M., de Chinaglia K.O., de Campos E.G., Costa J.L. (2021). Dispersive Liquid–Liquid Microextraction of 11-nor-Δ9-Tetrahydrocannabinol-Carboxylic Acid Applied to Urine Testing. Bioanalysis.

[68] Danila G.M., Puiu M., Zamfir L.G., Bala C. (2022). Early Detection of Cannabinoids in Biological Samples Based on Their Affinity Interaction with the Growth Hormone Secretagogue Receptor. Talanta.

[69] Borden S.A., Saatchi A., Palaty J., Gill C.G. (2022). A Direct Mass Spectrometry Method for Cannabinoid Quantitation in Urine and Oral Fluid Utilizing Reactive Paper Spray Ionization. Analyst.

[70] Gerace E., Bakanova S.P., Di Corcia D., Salomone A., Vincenti M. (2021). Determination of Cannabinoids in Urine, Oral Fluid and Hair Samples after Repeated Intake of CBD-Rich Cannabis by Smoking. Forensic Sci. Int..

[71] Sartore D.M., Vargas Medina D.A., Costa J.L., Lanças F.M., Santos-Neto Á.J. (2020). Automated Microextraction by Packed Sorbent of Cannabinoids from Human Urine Using a Lab-Made Device Packed with Molecularly Imprinted Polymer. Talanta.

[72] Samyn N. (2022). Mentor Article: Nele Samyn: Oral Fluid Testing of Drugged Drivers: My 25-Year Experience with an Interesting but Challenging Alternative Matrix. TIAFT Bull..

[73] Drummer O.H. (2006). Drug Testing in Oral Fluid Olaf. Clin. Biochem. Rev..

[74] Molnar A., Lewis J., Doble P., Hansen G., Prolov T., Fu S. (2012). A Rapid and Sensitive Method for the Identification of Delta-9-Tetrahydrocannabinol in Oral Fluid by Liquid Chromatography-Tandem Mass Spectrometry. Forensic Sci. Int..

[75] Ameline A., Gheddar L., Gaulier J.-M., Brunet B., Labat L., Eysseric H., Kintz P. (2022). Oral Fluid Concentrations of Drugs of Abuse: Interpretation Guide Proposed by the French Society of Analytical Toxicology for Road Traffic Safety. TIAFT Bull..

[76] Hoffman M.A., Hubbard J.A., Sobolesky P.M., Smith B.E., Suhandynata R.T., Sanford S., Sones E.G., Ellis S., Umlauf A., Huestis M.A. (2021). Blood and Oral Fluid Cannabinoid Profiles of Frequent and Occasional Cannabis Smokers. J. Anal. Toxicol..

[77] Wille S.M.R., Di Fazio V., del Mar Ramírez-Fernandez M., Kummer N., Samyn N. (2013). Driving Under the Influence of Cannabis: Pitfalls, Validation, and Quality Control of a UPLC-MS/MS Method for the Quantification of Tetrahydrocannabinol in Oral Fluid Collected With StatSure, Quantisal, or Certus Collector. Ther. Drug Monit..

[78] Niedbala S., Kardos K., Salamone S., Fritch D., Bronsgeest M., Cone E.J. (2004). Passive Cannabis Smoke Exposure and Oral Fluid Testing. J. Anal. Toxicol..

[79] Lin L., Amaratunga P., Reed J., Huang P., Lemberg B.L., Lemberg D. (2022). Quantitation of Δ8-THC, Δ9-THC, Cannabidiol and 10 Other Cannabinoids and Metabolites in Oral Fluid by HPLC-MS-MS. J. Anal. Toxicol..

[80] Andrews R., Paterson S. (2012). Production of Identical Retention Times and Mass Spectra for Δ9-Tetrahydrocannabinol and Cannabidiol Following Derivatization with Trifluoracetic Anhydride with 1,1,1,3,3,3-Hexafluoroisopropanol. J. Anal. Toxicol..

[81] Hart D. Cannabinoid Metabolites Pose Analytical Challenges in Urine Drug Testing Laboratories. https://www.dropbox.com/s/sycrf6ovxrbzkfp/NLCP_DTM_CBDA_Delta8THCA_Challenges_Hart_22Nov2019.pdf?dl=0.

[82] Golombek P., Müller M., Barthlott I., Sproll C., Lachenmeier D.W. (2020). Conversion of Cannabidiol (CBD) into Psychotropic Cannabinoids Including Tetrahydrocannabinol (THC): A Controversy in the Scientific Literature. Toxics.

[83] Coulter C., Wagner J.R. (2021). Cannabinoids in Oral Fluid: Limiting Potential Sources of Cannabidiol Conversion to Δ9- And Δ8-Tetrahydrocannabinol. J. Anal. Toxicol..

[84] Gorziza R., Cox J., Limberger R.P., Arroyo-Mora L.E. (2021). Study of Δ9-Tetrahydrocannabinol (THC) and Cannabidiol (CBD) Extraction FROM Dried Oral Fluid Spots (DOFS) and LC–MS/MS Detection. J. Cannabis Res..

[85] Mercier B., Scala-Bertola J., Pape E., Kolodziej A., Gibaja V., Bisch M., Jouzeau J.Y., Gambier N. (2022). Online SPE UPLC-MS/MS Method for the Simultaneous Determination of 33 Psychoactive Drugs from Swab-Collected Human Oral Fluid Samples. Anal. Bioanal. Chem..

[86] Coulter C., Garnier M., Moore C. (2022). Rapid Extraction and Qualitative Screening of 30 Drugs in Oral Fluid at Concentrations Recommended for the Investigation of DUID Cases. J. Anal. Toxicol..

[87] da Cunha K.F., Oliveira K.D., Huestis M.A., Costa J.L. (2020). Screening of 104 New Psychoactive Substances (NPS) and Other Drugs of Abuse in Oral Fluid by LC–MS-MS. J. Anal. Toxicol..

[88] Barroso M., Gallardo E., Vieira D.N., López-Rivadulla M., Queiroz J.A. (2011). Hair: A Complementary Source of Bioanalytical Information in Forensic Toxicology. Bioanalysis.

[89] Dulaurent S., Gaulier J.M., Imbert L., Morla A., Lachâtre G. (2014). Simultaneous Determination of Δ9-Tetrahydrocannabinol, Cannabidiol, Cannabinol and 11-nor-Δ9-Tetrahydrocannabinol-9-Carboxylic Acid in Hair Using Liquid Chromatography-Tandem Mass Spectrometry. Forensic Sci. Int..

[90] Thieme D., Sachs H., Uhl M. (2014). Proof of Cannabis Administration by Sensitive Detection of 11-nor-Delta(9)-Tetrahydrocannabinol-9-Carboxylic Acid in Hair Using Selective Methylation and Application of Liquid Chromatography- Tandem and Multistage Mass Spectrometry. Drug Test. Anal..

[91] Kuwayama K., Miyaguchi H., Yamamuro T., Tsujikawa K., Kanamori T., Iwata Y.T., Inoue H. (2015). Micro-Pulverized Extraction Pretreatment for Highly Sensitive Analysis of 11-nor-9-Carboxy-Δ9-Tetrahydrocannabinol in Hair by Liquid Chromatography/Tandem Mass Spectrometry. Rapid Commun. Mass Spectrom..

[92] Park M., Kim J., Park Y., In S., Kim E., Park Y. (2014). Quantitative Determination of 11-nor-9-Carboxy-Tetrahydrocannabinol in Hair by Column Switching LC-ESI-MS3. J. Chromatogr. B Anal. Technol. Biomed. Life Sci..

[93] Hehet P., Franz T., Kunert N., Musshoff F. (2022). Fast and Highly Sensitive Determination of Tetrahydrocannabinol (THC) Metabolites in Hair Using Liquid Chromatography-Multistage Mass Spectrometry (LC–MS3). Drug Test. Anal..

[94] Lo Faro A.F., Venanzi B., Pilli G., Ripani U., Basile G., Pichini S., Busardò F.P. (2022). Ultra-High-Performance Liquid Chromatography-Tandem Mass Spectrometry Assay for Quantifying THC, CBD and Their Metabolites in Hair. Application to Patients Treated with Medical Cannabis. J. Pharm. Biomed. Anal..

[95] Cobo-Golpe M., De-Castro-Ríos A., Cruz A., López-Rivadulla M., Lendoiro E. (2021). Determination and Distribution of Cannabinoids in Nail and Hair Samples. J. Anal. Toxicol..

[96] Schaefer V.D., Müller V.V., de Lima Feltraco Lizot L., Hahn R.Z., Schneider A., Antunes M.V., Linden R. (2021). Sensitive Determination of 11-nor-9-Carboxy-Δ9-Tetrahydrocannabinol and Complementary Cannabinoids in Hair Using Alkaline Digestion and Mixed-Mode Solid Phase Extraction Followed by Liquid-Chromatography-Tandem Mass Spectrometry. Forensic Sci. Int..

[97] Concheiro M., Gutierrez F.M., Ocampo A., Lendoiro E., González-Colmenero E., Concheiro-Guisán A., Peñas-Silva P., Macías-Cortiña M., Cruz-Landeira A., López-Rivadulla M. (2021). Assessment of Biological Matrices for the Detection of in Utero Cannabis Exposure. Drug Test. Anal..

[98] Mannocchi G., Di Trana A., Tini A., Zaami S., Gottardi M., Pichini S., Busardò F.P. (2020). Development and Validation of Fast UHPLC-MS/MS Screening Method for 87 NPS and 32 Other Drugs of Abuse in Hair and Nails: Application to Real Cases. Anal. Bioanal. Chem..

[99] Shin Y., Kim J.Y., Cheong J.C., Kim J.H., Kim J.H., Lee H.S. (2020). Liquid Chromatography-High Resolution Mass Spectrometry for the Determination of Three Cannabinoids, Two (−)-Trans-Δ9-Tetrahydrocannabinol Metabolites, and Six Amphetamine-Type Stimulants in Human Hair. J. Chromatogr. B Anal. Technol. Biomed. Life Sci..

[100] Hudson M., Stuchinskaya T., Ramma S., Patel J., Sievers C., Goetz S., Hines S., Menzies E., Russell D.A. (2019). Drug Screening Using the Sweat of a Fingerprint: Lateral Flow Detection of ’ " 9 -Tetrahydrocannabinol, Cocaine, Opiates and Amphetamine. J. Anal. Toxicol..

[101] Wurz G.T., Montoya E., DeGregorio M.W. (2022). Examining Impairment and Kinetic Patterns Associated with Recent Use of Hemp-Derived Δ8-Tetrahydrocannabinol: Case Studies. J. Cannabis Res..

[102] Luo Y.R., Yun C., Lynch K.L. (2019). Quantitation of Cannabinoids in Breath Samples Using a Novel Derivatization Lc-Ms/Ms Assay with Ultra-High Sensitivity. J. Anal. Toxicol..

[103] Dutkiewicz E.P., Urban P.L. (2016). Quantitative Mass Spectrometry of Unconventional Human Biological Matrices. Philos. Trans. R. Soc. A Math. Phys. Eng. Sci..

[104] Al-Asmari A.I. (2019). Method for Postmortem Quantification of Δ9-Tetrahydrocannabinol and Metabolites Using LC-MS-MS. J. Anal. Toxicol..

[105] Fabritius M., Staub C., Mangin P., Giroud C. (2012). Distribution of Free and Conjugated Cannabinoids in Human Bile Samples. Forensic Sci. Int..

[106] Pettersen S., Øiestad Å.M.L., Rogde S., Brochmann G.W., Øiestad E.L., Vindenes V. (2021). Distribution of Tetrahydrocannabinol and Cannabidiol in Several Different Postmortem Matrices. Forensic Sci. Int..

[107] Nicolaou A.G., Stavrou I.J., Louppis A.P., Constantinou M.S., Kapnissi-Christodoulou C. (2021). Application of an Ultra-Performance Liquid Chromatography-Tandem Mass Spectrometric Method for the Detection and Quantification of Cannabis in Cerumen Samples. J. Chromatogr. A.

[108] Chittamma A., Marin S.J., Williams J.A., Clark C., McMillin G.A. (2013). Detection of in Utero Marijuana Exposure by GC–MS, Ultra-Sensitive ELISA and LC–TOF–MS Using Umbilical Cord Tissue. J. Anal. Toxicol..

[109] Concheiro M., Huestis M.A. (2018). Drug Exposure during Pregnancy: Analytical Methods and Toxicological Findings. Bioanalysis.

[110] Lamy S., Hennart B., Houivet E., Dulaurent S., Delavenne H., Benichou J., Allorge D., Marret S., Thibaut F. (2017). Assessment of Tobacco, Alcohol and Cannabinoid Metabolites in 645 Meconium Samples of Newborns Compared to Maternal Self-Reports. J. Psychiatr. Res..

[111] Jensen T.L., Wu F., McMillin G.A. (2019). Detection of in Utero Exposure to Cannabis in Paired Umbilical Cord Tissue and Meconium by Liquid Chromatography-Tandem Mass Spectrometry. Clin. Mass Spectrom..

[112] Wu F., Jensen T.L., McMillin G.A. (2019). Detection of In Utero Cannabis Exposure in Umbilical Cord Tissue by a Sensitive Liquid Chromatography-Tandem Mass Spectrometry Method. LC-MS in Drug Analysis: Methods and Protocols.

[113] Sempio C., Wymore E., Palmer C., Bunik M., Henthorn T.K., Christians U., Klawitter J. (2021). Detection of Cannabinoids by LC-MS-MS and ELISA in Breast Milk. J. Anal. Toxicol..

[114] Ramnarine R.S., Poklis J.L., Wolf C.E. (2019). Determination of Cannabinoids in Breast Milk Using QuEChERS and Ultra-Performance Liquid Chromatography and Tandem Mass Spectrometry. J. Anal. Toxicol..

[115] Liu P., Liu W., Qiao H., Jiang S., Wang Y., Chen J., Su M., Di B. (2022). Simultaneous Quantification of 106 Drugs or Their Metabolites in Nail Samples by UPLC-MS/MS with High-Throughput Sample Preparation: Application to 294 Real Cases. Anal. Chim. Acta.

[116] Busardò F.P., Gottardi M., Pacifici R., Varì M.R., Tini A., Volpe A.R., Giorgetti R., Pichini S. (2020). Nails Analysis for Drugs Used in the Context of Chemsex: A Pilot Study. J. Anal. Toxicol..

[117] Hernandez A., Lacroze V., Doudka N., Becam J., Pourriere-Fabiani C., Lacarelle B., Solas C., Fabresse N. (2022). Determination of Prenatal Substance Exposure Using Meconium and Orbitrap Mass Spectrometry. Toxics.

[118] de Carvalho Mantovani C., e Silva J.P., Forster G., de Almeida R.M., de Albuquerque Diniz E.M., Yonamine M. (2018). Simultaneous Accelerated Solvent Extraction and Hydrolysis of 11-nor-Δ9-Tetrahydrocannabinol-9-Carboxylic Acid Glucuronide in Meconium Samples for Gas Chromatography–Mass Spectrometry Analysis. J. Chromatogr. B Anal. Technol. Biomed. Life Sci..

[119] Metz T.D., Silver R.M., McMillin G.A., Allshouse A.A., Jensen T.L., Mansfield C., Heard K., Kinney G.L., Wymore E., Binswanger I.A. (2019). Prenatal Marijuana Use by Self-Report and Umbilical Cord Sampling in a State with Marijuana Legalization. Obstet. Gynecol..

[120] Kim J., de Castro A., Lendoiro E., Cruz-Landeira A., López-Rivadulla M., Concheiro M. (2018). Detection of in Utero Cannabis Exposure by Umbilical Cord Analysis. Drug Test. Anal..

[121] Wu F., Scroggin T.L., Metz T.D., McMillin G.A. (2018). Development of a Liquid Chromatography-Tandem Mass Spectrometry Method for the Simultaneous Determination of Four Cannabinoids in Umbilical Cord Tissue. J. Anal. Toxicol..

[122] Bertrand K.A., Hanan N.J., Honerkamp-Smith G., Best B.M., Chambers C.D. (2018). Marijuana Use by Breastfeeding Mothers and Cannabinoid Concentrations in Breast Milk. Pediatrics.

